# Road Accident Prevention with Instant Emergency Warning Message Dissemination in Vehicular Ad-Hoc Network

**DOI:** 10.1371/journal.pone.0143383

**Published:** 2015-12-04

**Authors:** Gokulakrishnan P, Ganeshkumar P

**Affiliations:** 1 Department of Computer Science and Engineering, PSNA College of Engineering and Technology, Dindigul – 624622, Tamilnadu, India; 2 Department of Information Technology, PSNA College of Engineering and Technology, Dindigul – 624622, Tamilnadu, India; Beihang University, CHINA

## Abstract

A Road Accident Prevention (RAP) scheme based on Vehicular Backbone Network (VBN) structure is proposed in this paper for Vehicular Ad-hoc Network (VANET). The RAP scheme attempts to prevent vehicles from highway road traffic accidents and thereby reduces death and injury rates. Once the possibility of an emergency situation (i.e. an accident) is predicted in advance, instantly RAP initiates a highway road traffic accident prevention scheme. The RAP scheme constitutes the following activities: (i) the Road Side Unit (RSU) constructs a Prediction Report (PR) based on the status of the vehicles and traffic in the highway roads, (ii) the RSU generates an Emergency Warning Message (EWM) based on an abnormal PR, (iii) the RSU forms a VBN structure and (iv) the RSU disseminates the EWM to the vehicles that holds the high Risk Factor (RF) and travels in High Risk Zone (HRZ). These vehicles might reside either within the RSU’s coverage area or outside RSU’s coverage area (reached using VBN structure). The RAP scheme improves the performance of EWM dissemination in terms of increase in notification and decrease in end-to-end delay. The RAP scheme also reduces infrastructure cost (number of RSUs) by formulating and deploying the VBN structure. The RAP scheme with VBN structure improves notification by 19 percent and end-to-end delay by 14.38 percent for a vehicle density of 160 vehicles. It is also proved from the simulation experiment that the performance of RAP scheme is promising in 4-lane highway roads.

## Introduction

The highway roads should be efficiently utilized for social and economical development of a nation. Insufficient infrastructure for transportation might slowdown the progress of a nation. The delay in transportation, increased traffic jams, raise in fuel cost, air pollution due to exhaust emissions and fuel shortage in future are very serious problems to address [1,2]. In this regard, the researchers took keen interest and arrived at different models such car following model, lattice hydrodynamic model, etc. [3,4] The World Health Organization (WHO) report says that above 1.24 million people die because of road traffic accidents. Globally, road traffic accidents are ranked as eighth cause of deaths and injuries [5]. In India, 4,43,001 road accidents have taken place and a total of 1,37,423 deaths and 4,94,893 injuries were reported during 2013 and it seems increasing by 1.4 percent every year [6]. In 2014, the global vehicle population is found to be 1.2 billion and is expected to reach 2 billion by the year 2035 [7]. The vehicle population has drastically increased over the past two decades and proportionally the highway road traffic has also increased [8]. This means that the number of road accidents increase every year throughout the world. These road accidents cause increase in death and injury rates and this in turn cause social and economic damage to the nation. In many developing countries like India, development of highway road infrastructure is not in proportion to the increase in vehicle population [9]. The highway infrastructure is poorer whereas most of the modern day’s vehicles are more user friendly, intelligent and safe for driving [1]. This forces the highway infrastructure to be more intelligent. Lot of research is required in this field to make highway infrastructure as feasible and viable for modern vehicles. Throughout the world more initiatives are taken for highway road traffic safety [10] in order to reduce road accidents and improve the injury and death rate.

The objective of this paper is to design a scheme for preventing highway road traffic accidents. A Road Accident Prevention (RAP) scheme is proposed in this paper for VANET to enable and enhance Intelligent Transportation System (ITS).

The highway road accidents are categorized as initial and secondary accidents [11, 12].The RAP scheme alerts the vehicles traveling in the road with an early emergency warning to prevent them from initial and secondary accidents. The RAP scheme uses both fixed Road Side Unit (RSU) in highway and the vehicles traveling in the highway, for accident prevention process. The RAP scheme aims to improve the performance of data dissemination in VANET by delivering the EWM to the vehicles needed within the stipulated time. Here, dissemination is the process of delivering messages from a source node to all other nodes in the current network [13, 14].

**Fig 1:**
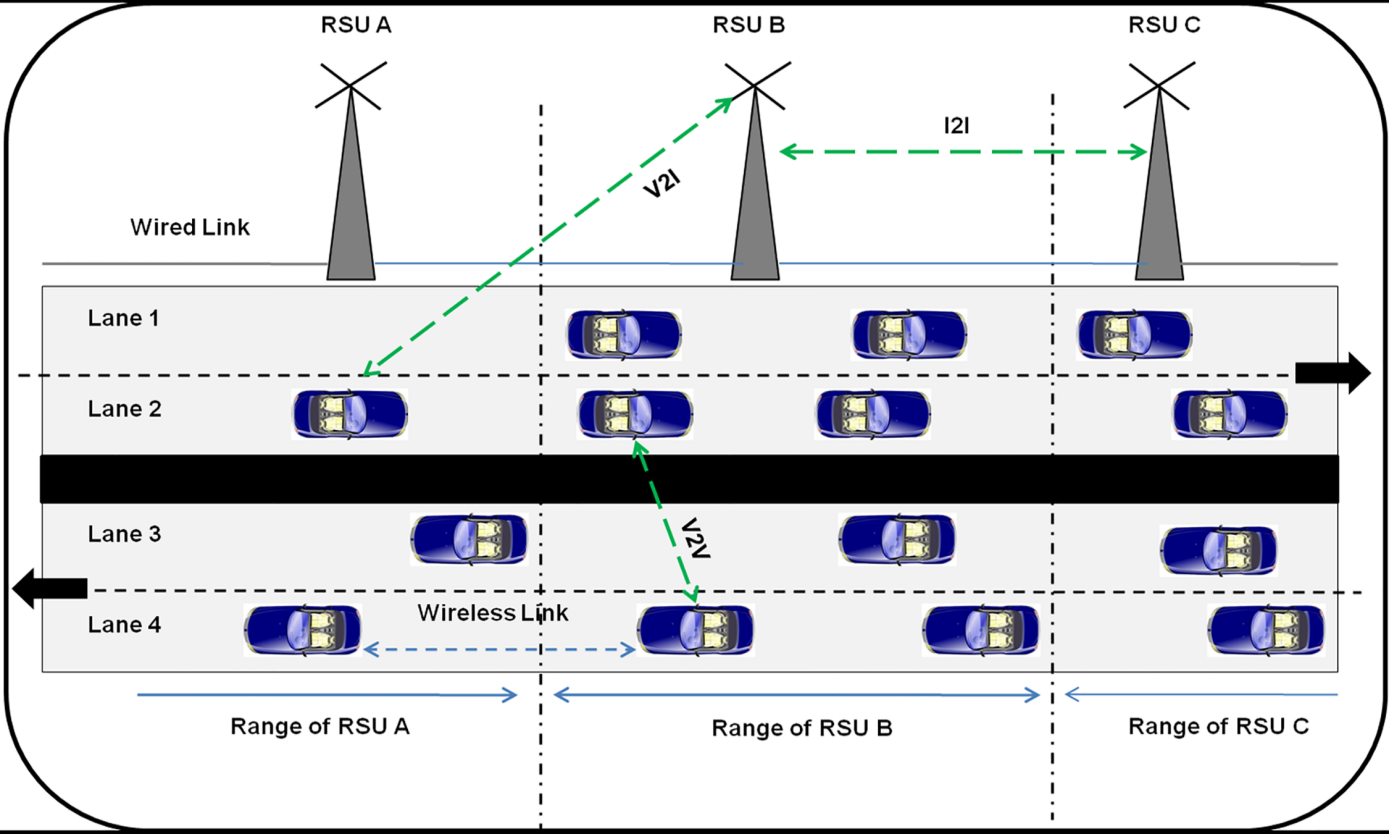
VANET Architecture.

In VANET, achieving high notification and low end-to-end delay are very difficult due to reasons such as dynamic vehicle density, high mobility of vehicles and limited bandwidth [15]. But in vehicle or road safety applications, robust and delay tolerant dissemination is a must [16, 17, 18] and this can be implemented with the features of delay tolerant network communication. The architecture of VANET is shown in Fig 1.

The RAP scheme is a supplementary scheme for highway road accident prevention. The actions of RAP scheme depend on some mechanisms which provide information about the possibility of occurrence of a highway road accident. In our previous work [19] Emergency Situation Prediction Mechanism (ESPM) performs prediction of emergency situation (ie a highway road accident) in highway roads and reports this to the near-by RSU. Based on this prediction information, the RAP scheme can initiate the prevention process. Before exercising the prevention process, certain questions need to be answered:

1. When to generate EWM and who will generate EWM?
2. What is the structure of EWM?
3. How to disseminate EWM to the vehicles in HRZ?

The RAP scheme addresses the above questions and offers following contributions: First, based on successful prediction of emergency situation in advance by using protocol like ESPM, the RSU generates an Emergency Warning Message (EWM). Second, the structure of EWM consists of information such as (i) EWM ID (ii) source (RSU) ID (iii) vehicle ID (iv) position of the emergency causing vehicle (v) speed of the vehicle and (vi) travel direction of the vehicle. Finally, the EWM is instantly disseminated to all the vehicles which hold the high Risk Factor (RF) and travel in High Risk Zone (HRZ). These vehicles travel both within and outside the RSU’s coverage (extending the coverage area of RSU by using the VBN structure). Suppose if the vehicles receive the EWM, they make necessary decisions such as slowing down, performing lane change, de-touring and choosing alternate routes [20]. The system and driver of the vehicle should cooperate with short reaction time for successful prevention of accidents [21]. The role of the driver in the road accident prevention is vital. The reaction time of the driver for acceleration or deceleration adjustment is strongly influenced by perturbations and traffic interruption probability [22, 23]. It is learned from literature that the dynamism in the traffic flow has an impact on the driver behavior. Further, the traffic interruption probability might make the traffic flow stable [24]. The fuel economy can be optimized via applications like Fuel Economy Optimization System (FEOS), which assists the driver to cut the fuel requirement during accelerations and decelerations [25, 26, 27].

The performance of RAP scheme is experimented by using simulation in Network Simulator (NS-2). The simulation results show that the RAP scheme is promising towards improvement in performance by dissemination of EWMs to the vehicles for highway road accident prevention.

Further, the paper is organized as follows: section 2 explains about the background, section 3 highlights the related system, the RAP design and methodology is discussed in section 4, section 5 and section 6 explain about simulation setup and results respectively, finally section 7 concludes.

## Background

In recent years, utmost importance is given to Intelligent Transportation System (ITS) for safe, efficient and comfort travel [28]. ITS is very much essential for social and economical development of nations. The Automated Highway System (AHS), Advanced Safety Vehicles (ASV) and Vehicle Infrastructure Integration (VII) make the transportation system to be much intelligent [1].

The VANET is a form of Mobile Ad-hoc Network (MANET) with only difference in movement of vehicular nodes. The movement of vehicles is based on traffic and traffic regulations. Hence in VANET, the movement of vehicles is regular [29]. In VANET, three types of communications are carried out for data or message transmissions such as Vehicle to Vehicle (V2V), Vehicle to Infrastructure (V2I) and Infrastructure to Infrastructure (I2I) [11, 30, 31, 32, 33, 34]. V2V communication is performed by using Dedicated Short Range Communication (DSRC), V2I communication is performed by using IEEE 802.11p and I2I communication is performed by using IEEE 802.3u.

In VANET, if proper message forwarding and networking mechanisms are not provided, then it might result in more packet collisions, leading to throughput degradation because of the broadcast storm problem [33, 35]. Single-hop broadcast might increase delay. Hence, a multi-hop broadcast protocol is required. The Multi-hop broadcast protocol is required for vehicles or road safety applications in VANET [11]. The successful dissemination of multi-hop warning messages beyond the transmission range of a vehicle faces three major issues such as broadcast storm problem, severe interference with existing periodic single-hop safety messages, and hidden nodes problem [36].

Most of the present day vehicles (assumed to be a car) are embedded with intelligent system of sensors, Global Positioning System (GPS) and transceivers for transmission and reception of signals [1, 37]. The vehicle closer to the accident area receives emergency messages and reacts according to the situation by either slowing down or performing lane change. The vehicles far away from the accident zone have a higher probability of reception of emergency messages and might take decisions such as detour or choosing alternate routes [38].

**Fig 2:**
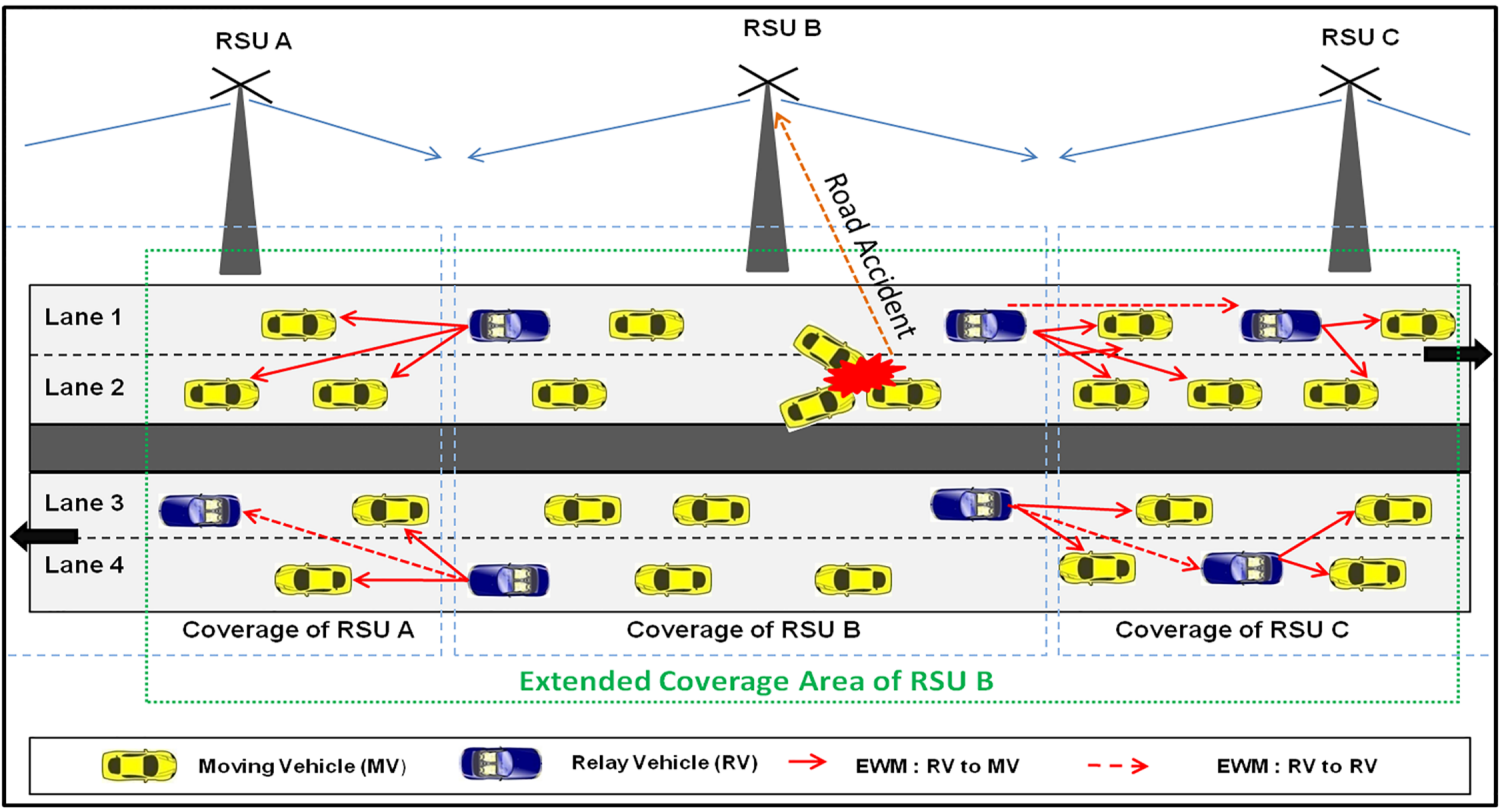
Organization of VBN Structure.

The Vehicular Backbone Network (VBN) is a VANET structure [39] that uses the road side units and vehicles as the members of the network. The VBN structure is developed from Mobile Backbone Network (MBN) by considering the movement of the vehicles along the linear highway road [40]. The organization of VBN is shown in Fig 2.

In VBN, particular vehicles are dynamically elected as Relay Nodes (RN) to act as base station. These relay nodes are used as backbone forwarding nodes for a specific period of time [20, 41]. In case if a relay node detects a path or link failure (loss of signals), then the relay node can choose an alternate path with a different set of intermediate nodes for emergency warning message dissemination [42–46].A vehicle is permitted to act as a relay node when it is within a specific distance limit from the road side unit. Once a vehicle goes away from the coverage of road side unit, a new vehicle closer to RSU will be reelected as RN. The RN selection procedure is explained in Relay Node (RN) Selection section.

The utilization of VBN structure has several advantages: (i) reduction in S-D distance (ii) decrease in end-to-end delay, (iii) increase in EWM notification, (iv) extension of the coverage area of the road side unit and (v) reduction in the number of RSUs required. This is experimented in simulation and a detailed discussion is presented in simulation setup and results and discussion sections.

Based on Indian four lane highways, the highway road considered in this paper consists of four lanes, two in each direction with a divider and follows the Indian traffic system (left hand traffic). These lanes are fixed with minimum speed limit of 60 km/h and maximum speed limit of 90 km/h [47]. If the vehicle travels at a speed of 60 km/h, then it resides within the coverage area of RSU (500 meters) for 30 seconds. On the contrary, if the vehicle travels at a speed of 90km/h, then it travels in the coverage area of RSU for a mere 19.8 seconds. In our previous work [19], the four lane highway is assumed to be embedded with sensors and it forms a Four Lane Sensor Grid (FLSG). The sensor coverage is found to be 6 meters and the vehicle is present within this area for 240 ms if it travels at a speed of 60 km/h. With respect to this, the periodic timer is set to the minimum value of 100ms for most of the control activities of the RAP scheme.

## Related Work

Junliang Liu et al [13] proposed an on-time warning delivery mechanism for VANET using IEEE 802.11p. This mechanism is based on receiver’s agreement on forwarding strategies in two dimensional vehicular networks. The results of simulation show that the number of collisions is lesser and the performance of reliability and delay are higher for warning message dissemination overriding broadcast storm problem.

Celimuge Wu et al [15] proposed a BackBone BRoadcast (BBBR) protocol for data dissemination in VANET with dynamic backbone selection. This protocol reduces the MAC contention time at each node with high data dissemination ratio. Both theoretical and simulation results show that the throughput and delay are improved.

Izhak Rubin et al [20] proposed a system for critical public safety message broadcasting. In this system, the RSU in highway broadcasts critical safety messages to the vehicles in its vicinity and to the vehicles over the vicinity using VBN. The authors used vehicular Carrier Sense Multiple Access/ Collision Avoidance (CSMA/CA) access scheme and spatial reuse Time Division Multiple Access (TDMA) to emulate the system. This system forwards the messages with high throughput and low end-to-end delay.

Muhammad Awais Javed et al [36] proposed a multi-hop broadcast protocol for emergency warning notification in highway VANET. This paper attempts to overcome broadcast storm, severe interference and hidden node problems. The results of simulation show that this protocol performs better in terms of number of multi-hop transmissions and dissemination delay.

Izhak Rubin et al [35] proposed a Lane Based Election (LBE) algorithm for the relay node selection by using VBN structure in highway VANET. This paper focuses on optimal selection of the relay nodes. This paper also proposes Group Based Election (GBE) algorithm which is used in case the lane residence information is not employed. The simulation results on analytical expressions show that these algorithms perform better for more number of lanes with varied vehicle densities.

Xing Fan et al [37] proposed a multi-hop broadcast scheme with RSU assistance. This scheme ensures in instant emergency message propagation. The authors express that the throughput and network utilization are better in this scheme irrespective of the number of vehicles.

Francesca Cuomo et al [39] proposed a protocol for dissemination scheme by using VANET structure called VBN for achieving high throughput. The authors presented both analytical and simulation analysis by using IEEE 802.11p CSMA/CA MAC protocol. The protocol achieves high throughput and low end-to-end delay. This approach is done for a linear highway and it needs to be extended for two dimensional highway systems.

Sok-Ian Sou et al [48] proposed a system that quantifies improvement in highway VANET connectivity with deployment of minimum number of RSUs. The authors have further investigated the performance of routing for broadcast based safety applications. Both analytical and simulation are done for performance comparison in terms of delay. This paper considers only linear highway and plans to consider two dimensional highway as a future work.

Pierpaolo Salvo et al [49] proposed a forwarding algorithm that extends the coverage area of RSU for Urban structures. The authors explained the limitation of RSU’s coverage with single-hop communication. They also impose importance on multi-hop and inter-vehicle communication for extending the coverage of RSU. The extension is made about 20 times greater than the original coverage area of RSU and it is noticed that only a few vehicles are not reached by the safety message. Only a few scenarios were taken into account and it has to consider many dynamic scenarios.

Jung-Chun Kao et al [50] proposed a WO-RANC protocol to enhance coverage and performance of IEEE 802.11p. WO-RANC is an ARQ protocol that uses relay assisted and work based wide sense opportunistic retransmission. The enhancement of coverage and performance is based on the relay nodes, which will forward and deliver the message on behalf of source node. The simulation shows that WO-RANC is better than IEEE 802.11p in terms of throughput and end-to-end delay for many Source–Destination distances.

Katsuhiro Naito et al [51] expressed about coverage extension of RSU with OFDM cooperative transmission. This paper proposed a range extension mechanism for RSUs in ITS networks. The simulation results show that this mechanism extends the range of RSU and achieves high throughput without degradation.

Yuan Yao et al [52] proposed a system for analyzing performance and reliability of IEEE 802.11p safety critical broadcast on the Control Channel (CCH) in Highway VANET. In this paper, the authors have proposed two markov chain models for analysis. The analytical and simulation results show that delay, packet reception rate and collision probability are better against various vehicle densities.

Behnam Hassanabadi and Shahrokh Valaee [53] proposed a system for reliable periodic safety message broadcasting in VANET. This system uses network coding algorithm and provides reliability for small safety messages with low overhead. Both analytical and simulation results show that the performance of this system is higher when compared to the previous schemes. This design can be integrated with Wireless Access in Vehicular Environment (WAVE) architecture.

Barłomiej Błaszczyszyn et al [54] proposed a system to maximize throughput in a linear highway VANET. The authors developed two models based on Signal to Noise Ratio (SINR). This system is instrumental in achieving maximum throughput by optimizing transmission range.Thus the performance of the network is improved.

Faika Hoque and Sungoh Kwon [55] proposed an efficient warning message forwarding scheme to avoid collisions. A multi-hop V2V communication and a two way intelligent broadcasting method is proposed. This approach attempts to avoid redundancy of warning messages and competeswith non emergency messages. The Simulation results show that the rear-end accidents are reduced by 70%. Further end-to-end delay is reduced by 55%.

Jiawei Huang et al [56] proposed Vehicle Density based Forwarding (VDF): a IEEE 802.11p based multi-hop protocol in VANET for emergency message dissemination. The simulation results show that the performance of VDF in case of end-to-end delay against vehicle density is improved.

Jae-In Choi et al [57] proposed a virtual slotted P-Persistence scheme in VANET for efficient and reliable dissemination of emergency messages. This scheme assures that the farthest vehicles will receive emergency message. The simulation results show that the performance of this scheme is better in terms of end-to-end delay, collision ratio and network overhead against vehicle density and distribution.

Hayder Salman Dawood and YumingWang [58] proposed an efficient broadcasting scheme for emergency messages in VANET. The area closer to the crashed vehicle is considered as the Risk Zone (RZ) and the emergency message is immediately broadcast to the vehicles in RZ using relay nodes. The results show that the performance of this protocol is better in terms of RZ Latency (RZL) and Beacons Overhead (BCO) against vehicle density.

Chakkaphong Suthaputchakun et al [59] proposed trinary partitioned black-burst-based broadcast protocol (3P3B): a multi-hop broadcasting protocol for time critical emergency message dissemination in VANET. The protocol uses IEEE 802.11p technology for communication. 3P3B protocol provides speedy and reliable emergency message dissemination by shortening channel access time and reducing contention period jitter. The results show that this protocol achieves better performance in terms of speed of message dissemination, communication delay and packet delivery ratio.

It is learned from the above works that (i) Multi-hop broadcasting with relay nodes in VBN structure in highway VANET will improve dissemination performance of time critical emergency warning messages. (ii) Extending the coverage area of RSU improves notification percentage and end-to-end delay. (iii) By extending the coverage area, the number of RSUs required is minimized. (iv) Reliability of EWM broadcasting is improved. (v) Reception of EWM by farthest vehicles is assured. Hence, in RAP scheme, the VBN structure is used for coverage extension and attempts are made to improve the performance of EWM dissemination in terms of notification percentage, end-to-end delay and reduction of the number of RSUs.

## RAP: Design and Methodology

**Fig 3:**
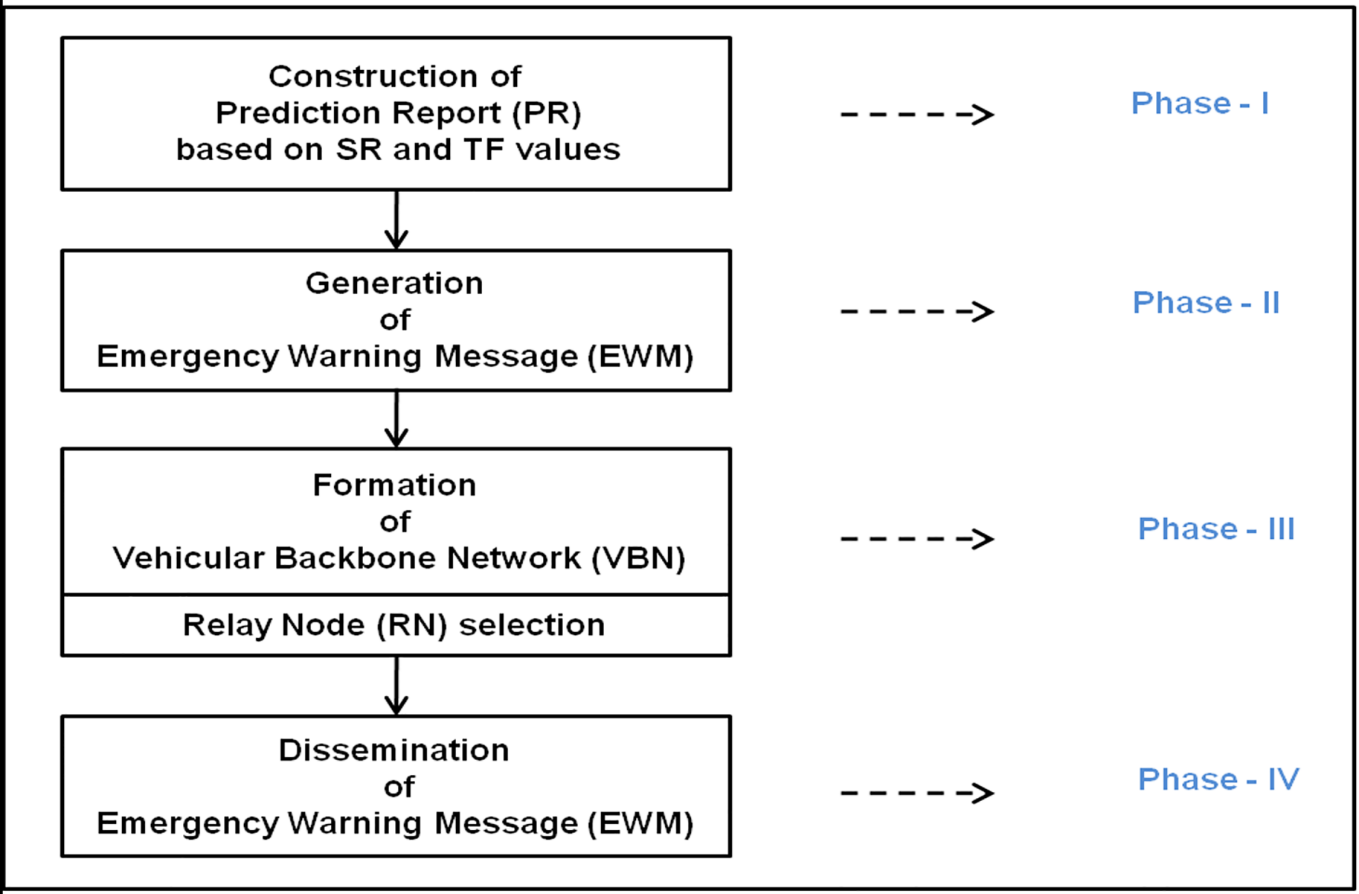
Overall structure of RAP scheme.

The proposed Road Accident Prevention (RAP) scheme is designed for preventing of highway road traffic accidents in Indian four lane express highways. The overall structure of RAP scheme is presented in Fig 3.

It is proved [60] that if the driver of the vehicle is informed about the occurrence of an accident in advance, then the driver might take precautionary actions to prevent the vehicle from road accident. Based on our previous work [19] it is known that if the possibility of a road accident is predicted in advance, then it would be easier to perform prevention.

The design of RAP scheme constitutes four phases such as (i) Prediction Report (PR) construction phase (ii) Emergency Warning Message (EWM) generation phase (iii) Vehicular Backbone Network (VBN) formation phase and (iv) EWM dissemination phase. The pseudo code of the RAP scheme is presented in following section. In the RAP scheme, the RSU constructs a Prediction Report (PR) based on abnormal Status Report (SR) and Traffic Flow (TF) data. Once the PR report is found to be abnormal then the RSU generates a EWM. The RSU forms a VBN structure and disseminates the EWM to the vehicles in High Risk Zone (HRZ) and to the vehicles in high risk factor.

### Pseudocode of RAP

**Definitions**:

EWM: Emergency Warning Message
VID: Vehicle ID
VPos: Position of the Vehicle
VSp: Speed of the Vehicle
YR: Yaw Rate
Vpres: Vehicle’s Presence
EWM ID: EWM‘s unique identity
VDir: Direction of the vehicle
SID: Source ID (ie RSU ID
SR: Status Report
TF: Traffic Flow
PR: Prediction Report
TD_val_: Travel Direction value
H-RN: Home—Relay Node
F-RN: Foreign—Relay Node
RSU-SR reg: SR register in RSU
RSU-TF reg: TF register in RSU
RSU-EWM reg: EWM register in RSU
RSU-VBN reg: VBN register in RSU
HRZ: High Risk Zone—Register
MRZ: Medium Risk Zone–Register
LRZ: Low Risk Zone—Register
NRZ: Non Risk Zone—Register
RF: Risk Factor (Low = 0, High = 3)
RP: Reception Priority (Low = 1, High = 2)

I. PR Construction Phase:

Module 1: (SR-Reporting Module)

1 Sleep until the *SRR_timer* expires
  1 For each vehicle (VID) on the road segment do:
    1 Read VSp, Vpos and YR from the vehicles.
    2 Construct SR.
    3 Store SR with VID in RSU-SR reg.
    4 Return.

Module 2: (TF-Reporting Module)

1 Sleep until the *TFR_timer* expires
  1 For each sensor_i_ in FLSG do:
    1 Receive Vpres.
    2 If (Vpres_t_ -Vpres_t-1_) = 0 for a sensor_i_.
      1 Set TF as 1.
      Else
      1 Set TF as 0.
    3 Store TF withSID in RSU-TF reg_._
    4 Return.

Module 3: (PR Construction Module)

Sub Module 1: (PR Construction based on SR)

1 Sleep until the *PRC1_timer* expires
  1 For each VID in RSU-SR reg do:
    1 Retrieve SR.
    2 If ((VSp_t_—VSp_t-1_> 30 km/h.) or (VSp_t-1_—VSp_t_< 30 km/h.) or (Vpos_t_—Vpos_t-1_ = 0) or (YR_t_—YR_t-1_> 30 degree) or (YR_t-1_—YR_t_< 30 degree)) then
      1 Set PR as abnormal.
      2 Store PR in RSU-PR reg.
      3 Return.

Sub Module 2: (PR Construction based on TF)

1 Sleep until the *PRC2_timer* expires
  1 For each SID in RSU-TF reg do:
    1 Retrieve TF.
    2 If (TF_t_ = TF_t-1_) for same SID then
      1 Set PR as abnormal.
      2 Store PR in RSU-PR reg.
      3 Return.

II. EWM Generation Phase:

1 Sleep until the *GEN_timer* (say 100 ms) expires
  1 Retrieve PR from RSU-PR reg.
  2 If the PR is Abnormal.
    1 Generate EWM with EWM ID, SID, VID, VPos, VSp and VDir.
    2 Store EWM in RSU-EWM reg.
    3 Return.

III. VBN Formation Phase:

Module 1: (Vehicle Selection)

Sub Module 1: (Selection of Vehicles within RSU coverage)

1 Sleep until the *VBN_timer*(say 100 ms) expires
  1 For each vehicle within RSU coverage do:
    1 If a vehicle enters into the range of RSU:
      1 Check VID in RSU-SR reg.
        1 If VID is not in RSU-SR reg.
          1 Add VID of the vehicle to RSU-VBN reg.
      Else If a vehicle leaves from the coverage of RSU:
      1 Remove VID of the vehicle from RSU-VBN reg.
    2 Select VID in RSU-VBN reg to act as H-RN.
      1 Call RN-Selection Algorithm.
  2 Return.

Sub Module 2: (Selection of Vehicles outside RSU coverage)

1 Sleep until the *VBN_timer*(say 100 ms) expires
  1 For each vehicle outside RSU coverage do:
    1 If a vehicle enters into the range of Foreign-RSU.
      1 Check VID in F-RSU-SR reg.
        1 If VID is not in F-RSU-SR reg.
          1 Add VID of the vehicle to F-RSU-SR reg.
      Else If a vehicle leaves from the range of RSU.
      1 Remove VID of the vehicle from F-RSU-SR reg.
    2 Update content of F-RSU-SR reg with RSU-VBN reg.
    3 Select VID in RSU-VBN reg to act as F-RN.
      3 Call RN-Selection Algorithm.
2 Return.

Module 2: (RN Selection)

1 Sleep until *VBN_ timer* (say 100ms expires)
  1 For each vehicle within RSU do:
    1 Retrieve VSp of the vehicle from RSU-VBN reg.
    2 Retrieve VPos of the vehicle from RSU-VBN reg.
    3 If VSp (VID) <60 km/h and VPos(VID) is within 20 meters to the coverage border.
      1 Select VID as H-RN.
  2 For each vehicle outside RSU coverage do:
    1 Retrieve VSp of the vehicle from RSU-VBN reg.
    2 Retrieve VPos of the vehicle from RSU-VBN reg.
    3 If VSp (VID) <60 km/h and VPos(VID) is within 20 meters outside the coverage border:
      1 Select VID as F-RN.
      2 Return.

IV. EWM Dissemination Phase:

Module 1: (RZ Identification)

1 Sleep until the *DISMN_timer* (say 100 ms) expires.
  1 For each vehicle within RSU coverage do:
    1 Retrieve VPos of the vehicle from RSU- VBN reg.
    2 If VPos is in between 0 and 200 meters range of RSU.
      1 Add VID to HRZ-R.
      Else if VPos is in between 201 and 300 meters range of RSU.
      1 Add VID to MRZ-R.
      Else if VPos is in between 301 and 500 meters range of RSU.
      1 Add VID to LRZ-R.
      Else
      1 Add VID to NRZ-R.
  2 Return.

Module 2: (TD Identification)

1 Sleep until the *DISMN_timer* (say 100 ms)expires.
  1 For each VID in RSU- VBN reg do:
    1 Retrieve VPos _t_ and VPos _t-1_ fromRSU- VBN reg_._
    2 Compute TD _val =_ VPos _t_—VPos _t-1_.
    3 If TD _val_ is greater than zero.
      1 Add VID to HRZ.
      Else
      1 Add VID to MRZ or LRZ.
    4 Return.

Module 3: (RF Assignment)

1 Sleep until the *DISMN_timer* (say 100 ms) expires.
  1 For each VID in RSU-VBN reg do:
    1 If VID is in HRZ.
      1 Set RF (VID) = 3.
      Else If VID is in MRZ.
      1 Set RF (VID) = 2.
      Else If VID is in LRZ.
      1 Set RF (VID) = 1.
      Else.
      1 Set RF (VID) = 0.
  2 Return.

Module 4: (EWM Prioritization)

1 Sleep until the *DISMN_timer* (say 100 ms) expires.
  1 If RF > = 2.
    1 Set RP (VID) = 2.
    Else
    1 Set RP (VID) = 1.
  2 For each VID in RSU- VBN reg do:
    1 If RP(VID) = 2.
      1 Read EWM from RSU-EWM reg.
      2 Perform Instant EWM Dissemination to the vehicles.
  3 Return.

### Prediction Report (PR) Construction Phase

This phase is used for constructing a Prediction Report (PR) and is organized into three modules such as SR reporting module, TF reporting module and construction module. The pseudocode of these modules is presented in pseudocode of RAP section.

In SR reporting module, once the SRR timer (100 milliseconds) expires, the RSU will read speed (VSp), position (VPos) and Yaw-Rate (YR) of the vehicles under its control. This module constructs the Status Report (SR) along with respective vehicle ID (VID) and stores it in RSU-SR register as shown in Tables 1 and 2. The movement of vehicles and their status for two time slots (t and t+1) is shown in Tables 1 and 2 respectively.

**Table 1:** Status RSU-SR at time‘t’.

Vehicle ID (VID)	Speed-VSp (km/h)	Position-Vpos (meters)	Yaw Rate-YR (degree)	Timer (milliseconds)
9397	65	150	6	25000
7860	70	135	3	25000
3339	90	122	12	25000
9097	55	105	3	25000
7560	45	90	5	25000
1317	95	70	9	25000
4046	50	45	4	25000

**Table 2:** Status RSU-SR at time‘t+1’.

Vehicle ID (VID)	Speed-VSp (km/h)	Position-Vpos (meters)	Yaw Rate-YR (degree)	Timer (milliseconds)
9397	65	151.8	5	24900
7860	70	136.95	3	24900
3339	90	124.5	10	24900
9097	55	106.5	0	24900
7560	45	91.25	0	24900
1317	95	72.65	10	24900
4046	50	46.39	3	24900

It can be noticed from the Tables 1 and 2 that vehicle 9397 moves about 1.8 meters (ie 151.8–151) in 100 ms at a travel speed of 65 km/h. The timer is set as 25000 ms because, this is the average time a vehicle travels in 500 meters coverage of RSU (discussed in Vehicular Backbone Network (VBN) formation phase section).

The highway road segments are embedded with sensors as described in our previous work [19]. The TF reporting module receives the presence of the vehicle (Vpres) from these sensors periodically. The value of the TFR timer is fixed as 240 milliseconds. This timer value is fixed based on the maximum time that a vehicle is supposed to be present in the sensor area of FLSG. That is, if a vehicle travels at a speed (minimum speed is considered) of 60 km/h then it will be present within the sensor area (6 meters) for 240ms. If the difference in Vpres for two continuous time slots is equal to zero, then the Traffic Flow (TF) data for the specific sensor is set as 1. Similarly, the TF data is specified for all the sensors and stored along with sensor ID (SID) in RSU-TF register as shown in Tables 3 and 4.

**Table 3:** Status of RSU-TF register at time ‘t’.

Sensor ID (SID)	(1,1)	(1,2)	(1,3)	(1,4)	(1,5)	(1,6)	(2,1)	(2,2)	(2,3)	---	(4,4)	(4,5)	(4,6)
**Traffic Flow Data (1 or 0)**	1	**1**	0	0	0	0	**1**	**1**	1	---	0	1	0
**Timer (milliseonds)**	240	240	240	240	240	240	240	240	240	---	240	240	240

**Table 4:** Status of RSU-TF register at time ‘t+1’.

Sensor ID (SID)	(1,1)	(1,2)	(1,3)	(1,4)	(1,5)	(1,6)	(2,1)	(2,2)	(2,3)	---	(4,4)	(4,5)	(4,6)
**Traffic Flow Data (1 or 0)**	0	**1**	0	0	0	0	**1**	**1**	0	---	0	0	1
**Timer (milliseonds)**	240	240	240	240	240	240	240	240	240	---	240	240	240

It is noticed in Tables 3 and 4 that the sensors (2,1) and (2,2) have the same TF data ‘1’. This alarms an abnormal situation.

Next to the traffic flow reporting module is the construction module and it is divided into two sub-modules. First, the prediction report construction is done based on the status report of the vehicles once in every 100 milliseconds (ie PRC1 timer). This module retrieves the status report for each vehicle from the RSU-SR reg. As proved in our previous work [19],it sets the prediction reportas abnormal if any of the following cases arise:

Case 1: The speed of the vehicle in two continuous time slots varies by 30 km/h.
Case 2: The position of the vehicle remains the same in two continuous time slots.
Case 3: The change in Yaw-Rate of the vehicle in two continuous time slots varies by 30 degree.

Second, the prediction report construction is done based on the traffic flow data once in every 480 milliseconds (i.e. PRC2 timer). If the traffic flow data for a set of sensors (SIDs) remains 1 for two continuous time slots, then this module sets the prediction report as abnormal. The prediction report status is stored in RSU-PR register as shown in Table 5.

**Table 5:** Status of RSU- PR register at different time slots.

Timer (milliseconds)	100	100	100	100	100	100	100	100	100	---	100	100	100
**PR based on SR**	**1**	0	0	0	0	0	**1**	0	0	---	0	**1**	0
**PR based on TF**	0	0	0	0	**1**	0	0	0	0	---	0	0	0

The prediction report is constructed for every 100ms. But the PRC2 timer in the second sub-module is set to be 480ms. Hence, prediction report construction is done based on status report. Once the PRC1 timer expires, the prediction report construction is done based on TF, likewise once the PRC2 timer expires (almost equal to 5 PRC1 timeslots). A entry ‘1’ in RSU-PR register indicates as abnormal situation.

### Emergency Warning Message (EWM) Generation Phase

**Fig 4:**
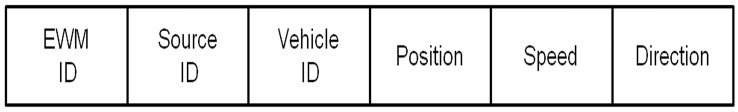
Structure of Emergency Warning Message.

This phase is instrumental in generating EWM based on the prediction report construction phase. This module retrieves the prediction report status from RSU-PR register once the GEN timer expires (100ms). With reference to the pseudocode of RAP section, if PR status is abnormal then the road side unit constructs an EWM. The Structure of the EWM is specified in Fig 4.

The EWM consists of: EWM ID which is used as EWM’s unique identity to avoid duplication, source ID is used for identification of RSU which is generating EWM, vehicle ID is used for identification of emergency causing vehicle, Position is the geographical position of the vehicle computed by using GPS, speed is the current speed of the vehicle sensed by speed sensor and direction is the moving direction of the vehicle computed by using two continuous position information.

### Vehicular Backbone Network (VBN) Formation Phase

**Fig 5:**
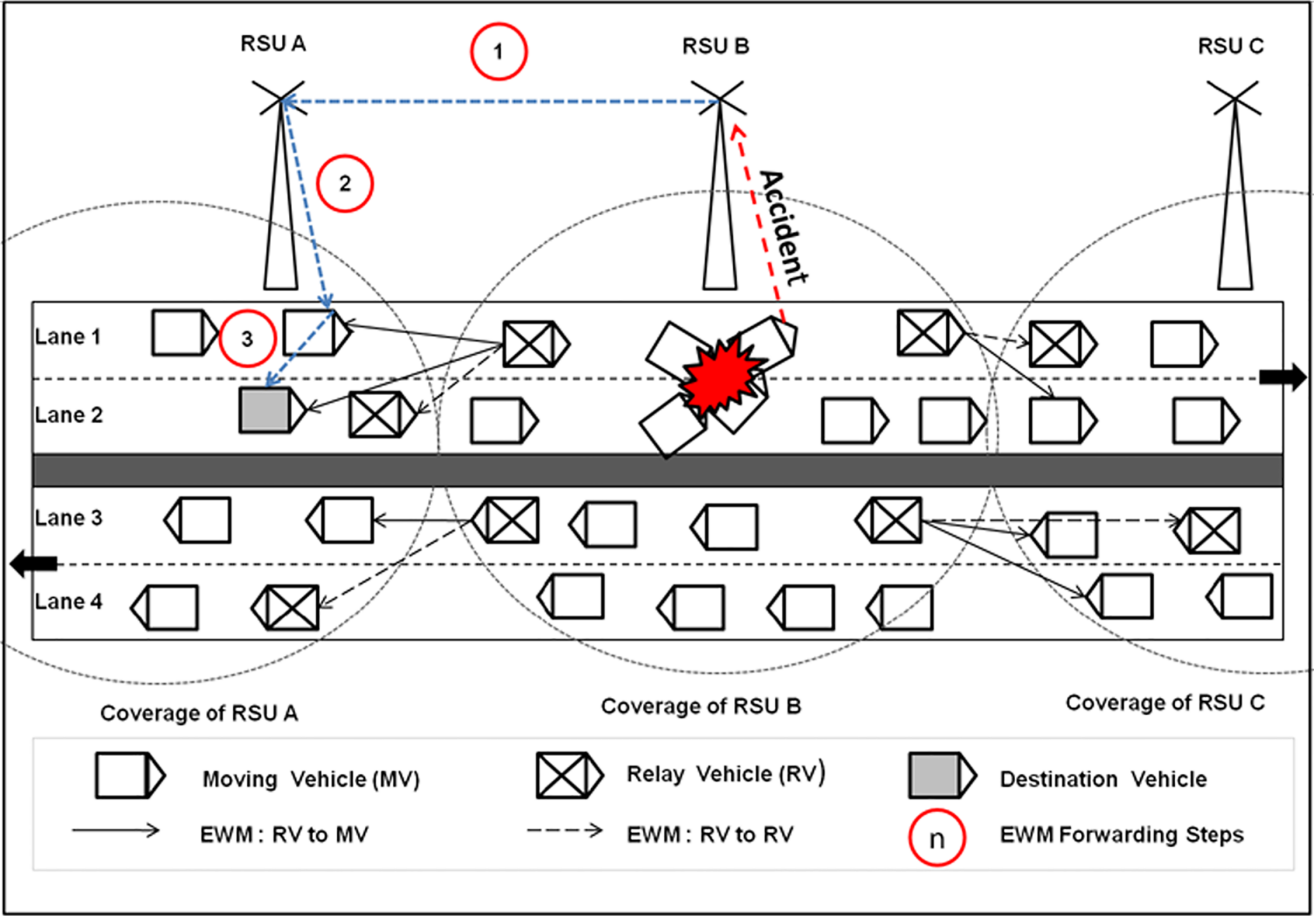
RAP Scheme 1: EWM Dissemination without VBN Structure.

**Fig 6:**
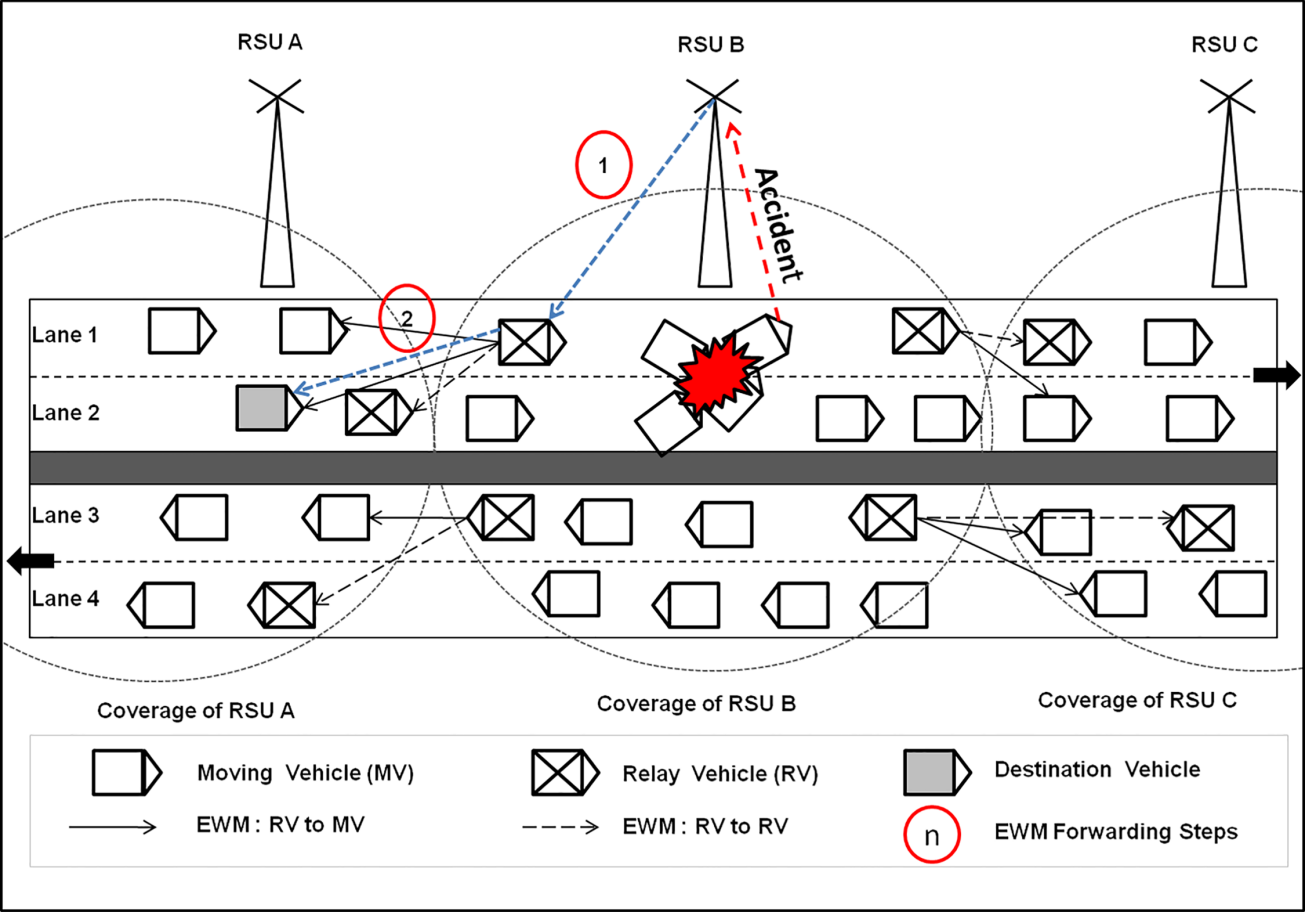
RAP Scheme 2: EWM Dissemination with VBN Structure.

Whenever the road side unit predicts an abnormality in highway road segment, broadcasting of EWM takes place in two steps. First, the road side unit broadcasts the EWM to all the vehicles in the area of coverage and to the near-by road side units. Second, the near-by road side units propagate EWM to the vehicles in their respective area of coverage. It is well known that the EWM should be delivered on time for prevention of road accidents. Let the second step be considered, where end-to-end delay would be poorer because the EWM has to travel a long distance to reach the destination as shown in Fig 5. Due to this problem, the VBN structure is introduced, where the EMW has to travel a lesser distance as shown in Fig 6.

The dissemination of EWM can be done by using two methods: First, the EWM can be disseminated without VBN relay nodes. Second, the EWM is disseminated to the destination vehicle with VBN relay nodes. In former, to reach a particular vehicle as shown in Fig 5, the S-D distance is found to be 550 meters. In later, the S-D distance is 300 meters as shown in Fig 6. By comparing the two methods, it is found that the use of VBN structure will definitely reduce the S-D distance. The S-D distance is mostly reduced via VBN structure due to coverage area extension of the RSU. The EWM is a time critical emergency message that has to be delivered in a short duration. Hence, the first method is not suitable and the second method that uses the VBN structure is adopted here. The methods discussed in related work section show that data dissemination using VBN structure yields high notification and low end-to-end delay.

The intention of VBN formation is to extend the coverage area of road side unit and there-by reduce the S-D distance for most of the vehicles and in turn reduce the number of road side units required. Irrespective of EWM broadcasting, the VBN structure is created dynamically once in every 100 milliseconds (VBN timer). The VBN structure is updated by making room for the incoming vehicles and negating the outgoing vehicles. The VBN formation phase is organized into two modules as vehicle selection module and relay node selection module and these modules are discussed in following sections.

#### Vehicle Selection

This vehicle selection module is used for selection of participating vehicles in VBN structure. This module is further divided into two sub modules for selection of vehicles to participate in the VBN structure. In first sub module, the vehicles entering into the RSU’s coverage are selected to participate in VBN structure. Once the vehicle enters into the coverage, the vehicle ID (VID) of the vehicle is checked in the RSU-SR register. If VID is not found in the RSU-SR register, then it is added to the RSU-VBN register. If the vehicle leaves the coverage area of road side unit, then the respective VID is deleted from RSU-VBN register. This process is repeated once in every 100ms (ie VBN timer expires).

In second sub module, the vehicles outside the road side unit’s coverage are selected to participate in the VBN structure. Similar to the first module, the incoming and the outgoing vehicles will be registered with near-by (or) foreign RSU-SR register (ie F-RSU-SR register). Once the VBN timer (100 ms)expires, this module updates the contents of foreign RSU-SR register with RSU-VBN register. By this way, the RSU-VBN register will have details of all the vehicles participating (both within and outside road side unit’s coverage) in the VBN structure.

The VBN structure would be complete only if certain participating vehicles are selected as the Relay Nodes (RN). These relay nodes are used to forward the EWM to the vehicles within road side unit’s coverage and the relay nodes outside the RSU’s coverage by using multi-hop communication. Any violation by the RNs in the forwarding process might make the entire system fail. Hence, the relay node selection algorithm should be much effective.

#### Relay Node (RN) Selection

In RAP scheme, two types of relay nodes are selected (i) the relay nodes that belongs to Source road side unit or Home RSU called Home RN (H-RN) and (ii) the relay nodes that belong to the near-by RSUs or Foreign-RSUs called Foreign RN (F-RN). In existing algorithms, the relay nodes are selected based on speed and distance between the vehicle and the RSU. But in RAP scheme, the relay nodes are selected based on criteria such as speed and position of the vehicle with respect to the road side unit’s coverage boundary.

**Fig 7:**
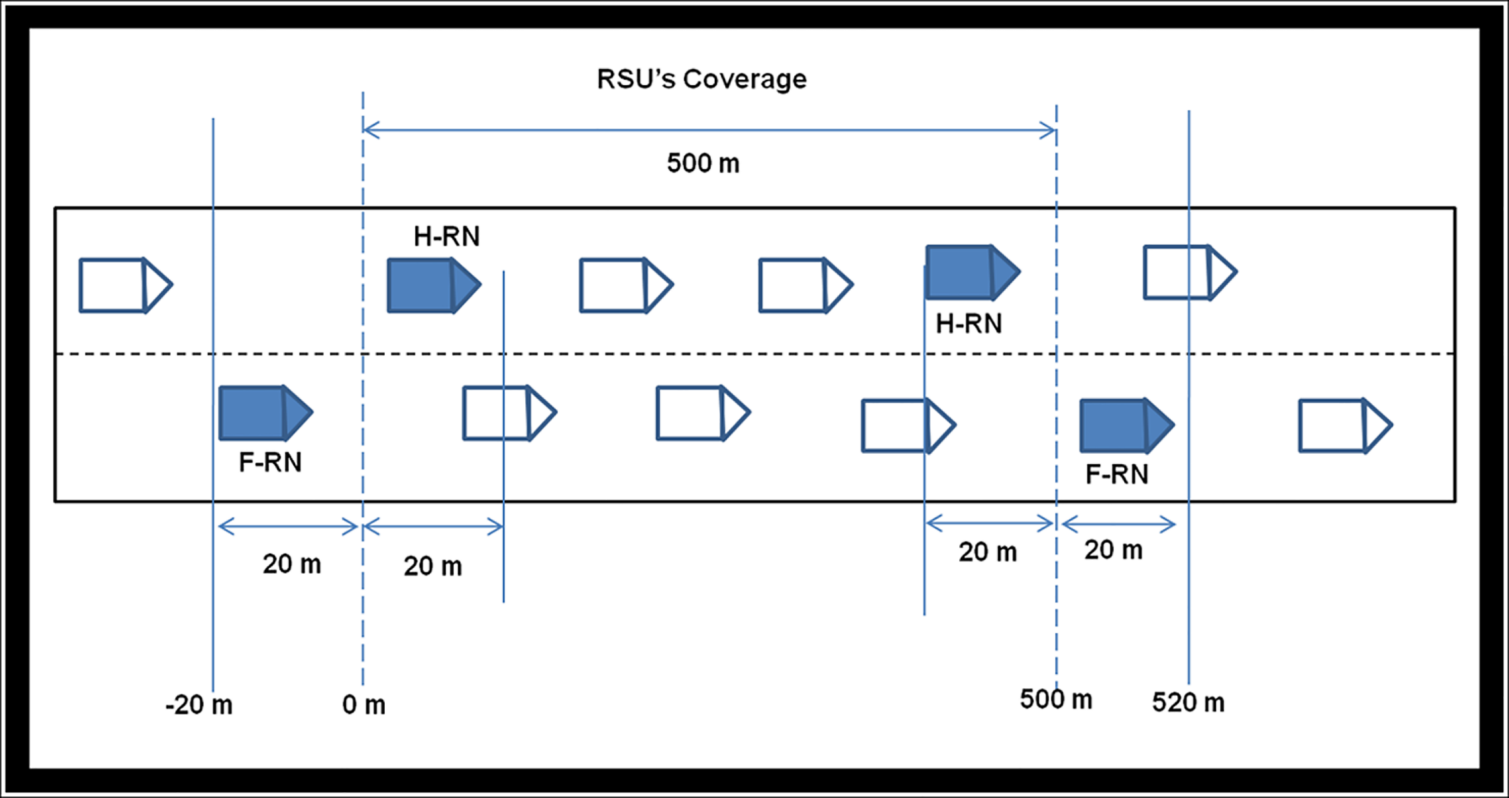
RN selection in VBN structure.

First criterion is chosen because a slow moving vehicle will reside in road side unit’s coverage for a longer time than a fast moving vehicle. That is the life time of a slow moving vehicle in the coverage area is more than other vehicles. Second criterion has been chosen because if the distance between H-RN and F-RN is lesser, then an effective EWM dissemination can be achieved. Once the VBN timer (100 ms) expires, this module retrieves speed and position of the vehicles from RSU-VBN register. As per Fig 7, if a vehicle’s speed is lesser than 60 km/h and the position of the vehicle lies either within 0 and 20 meters or 480 and 500 meters then that vehicle will be selected as H-RN.

In similar way, if the speed of the vehicle is lesser than 60 km/h and the position of the vehicle lies either within -20 and 0 or 500 and 520 meters, then the vehicle is selected as F-RN as shown in Table 6.The vehicles 1020 and 5007 are selected as H-RNs and the vehicles 8496 and 2129 are selected as F-RN, because these vehicles satisfy the criterion for RN selection.

**Table 6:** Status of RSU-VBN at time ‘t’.

Vehicle ID (VID)	Speed-VSp (km/h)	Position-Vpos (meters)	H-RN	F-RN	Timer (milliseconds)
1020	58	07	1	0	240
7651	70	135	0	0	240
5007	60	493	1	0	240
9237	95	105	0	0	240
8496	45	-12	0	1	240
2129	55	504	0	1	240
4652	65	45	0	0	240

### EWM Dissemination phase

The main objective of this phase is to deliver EWM instantly to all the vehicles closer to and far away from the Possible Accident Site (PAS) called Risk Zone (RZ). The pseudo code of this module is presented in pseudocode of RAP section. High priority should be given to vehicles which are travelling towards the PAS than to the vehicles which are traveling away from the PAS. This phase is organized into four modules such as (i) Risk Zone(RZ) identification module (ii) Travel Direction (TD) identification module (iii) Risk Factor (RF) assignment module and (iv) EWM prioritization module. The Dissemination (DISMN) timer in this phase is set as 100ms for all the control activities.

#### Risk Zone (RZ) Identification

**Fig 8:**
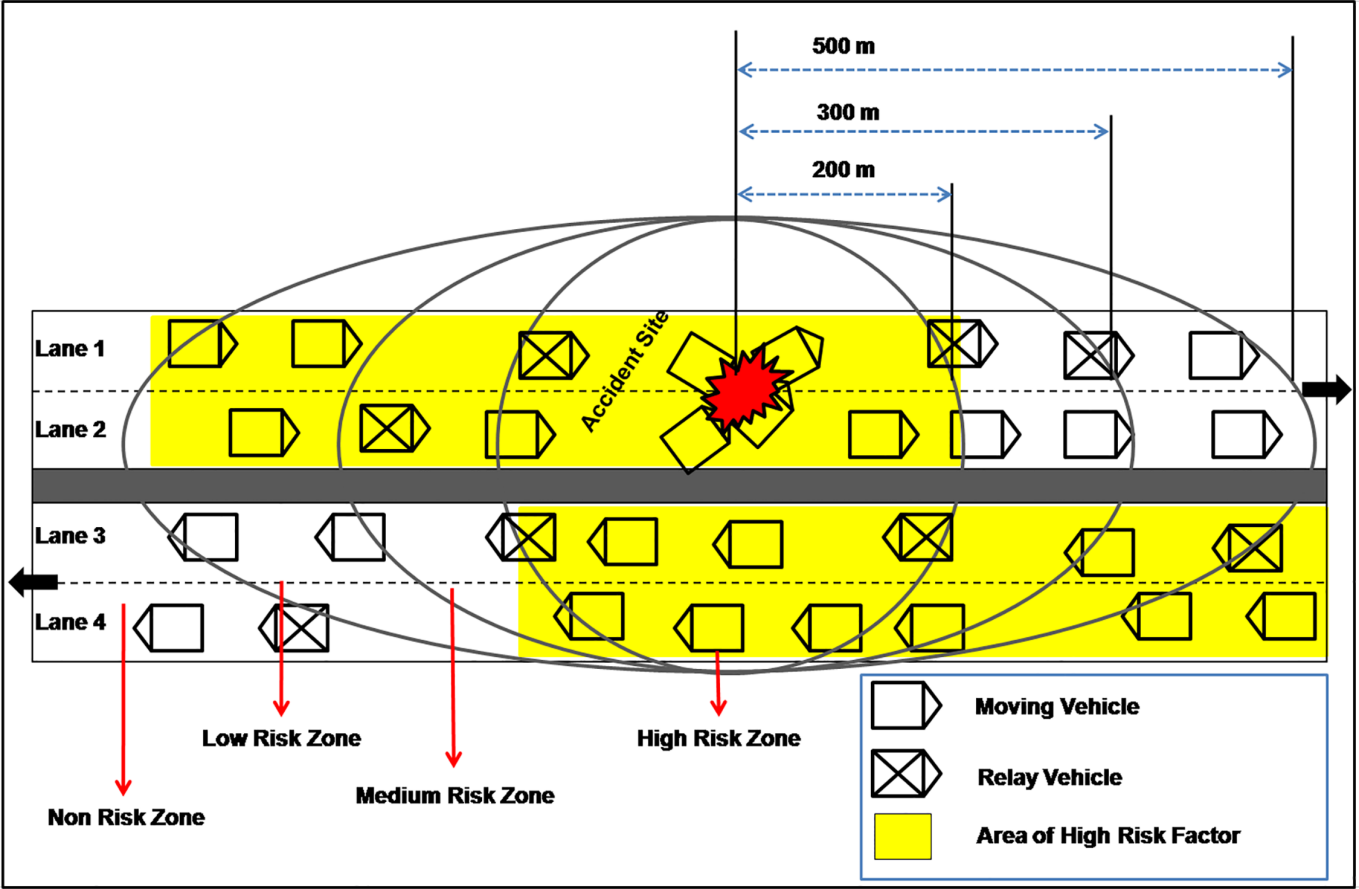
Representation of Risk Zones and Risk Factor.

The Risk Zone (RZ) is comprised of:(i) the possible vehicles involved in the accident (ii) surrounding or normal vehicles and (iii) the source road side unit. In RAP scheme, the Risk Zone is categorized into four types called High Risk Zone (HRZ), Medium Risk Zone (MRZ), Low Risk Zone (LRZ), and Non Risk Zone (NRZ)as shown in Fig 8.

**Fig 9:**
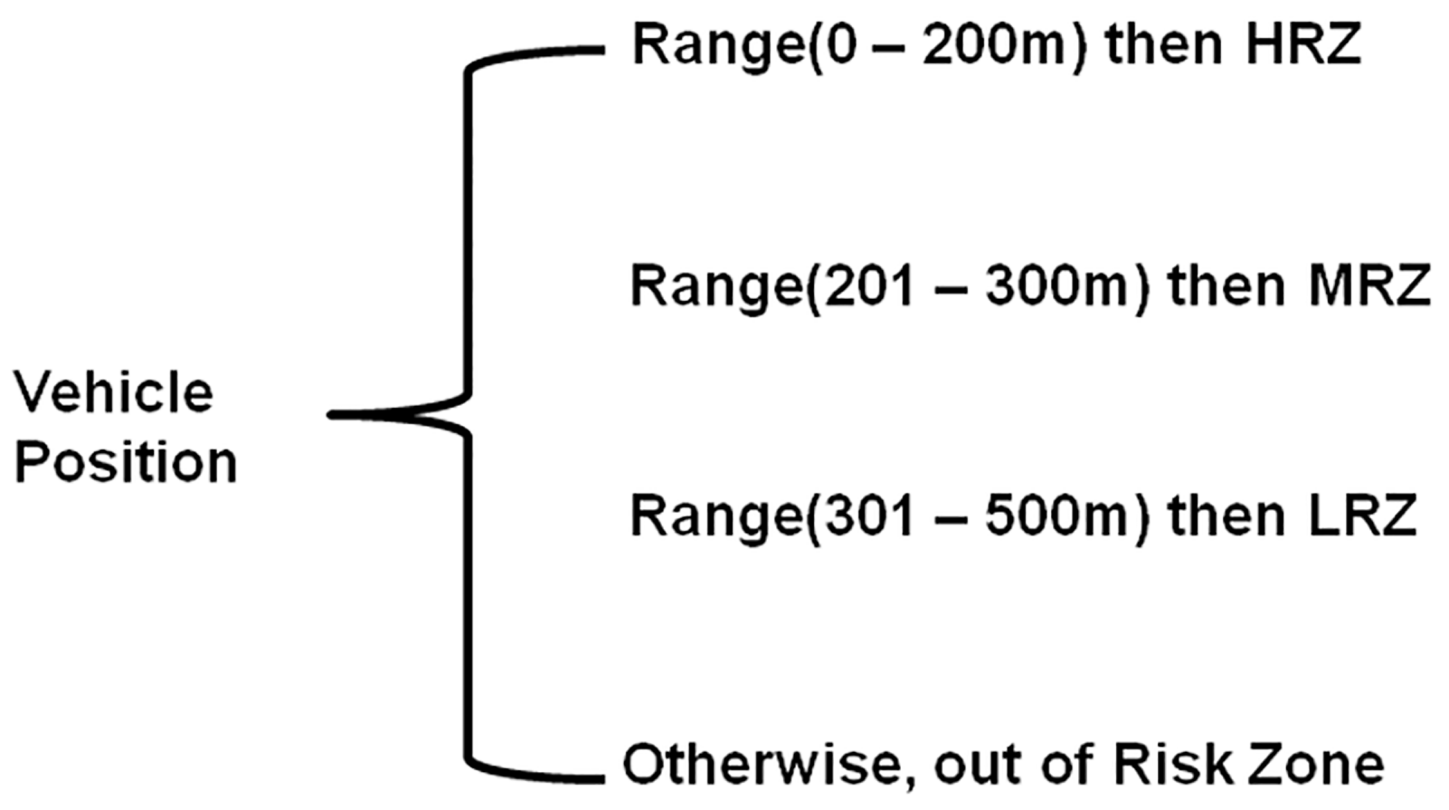
Risk Zones and ranges.

This module groups the vehicles (VIDs) of the VBN structure into risk zones based on the position (Vpos) of the vehicle retrieved from RSU-VBN register. The high risk zone is one where the surrounding or normal vehicles are much closer to the vehicles that might involve in accident (in PAS). As shown in Fig 9, the high risk zone range is fixed from0 to 200 meters. The low risk zone consists of vehicles which are farthest or faraway from the PAS. The range of low risk zone is fixed from301 to 500 meters. The medium risk zone lies in between high risk zone and low risk zone and the distance is fixed to be 201 to 300 meters from PAS. non risk zone is the zone beyond 500 meters from possible accident site.

The EWM should be immediately delivered to the vehicles in high risk zone as these vehicles have to instantly react either by slowing down or performing lane change. Next to high risk zone, the EWM delivery should be made to the vehicles in medium risk zone and then to the vehicles in low risk zone as these vehicles have to make a decision either to detour or take alternate routes to travel.

#### Travel Direction (TD) Identification

This module identifies the travel direction of a vehicle based on the Eq (1). The direction of travel of a vehicle is very important for risk factor assignment. The vehicle’s travel direction falls into two cases.

Case 1: Vehicles travelling towards the PAS, means that the vehicle is moving from low risk zone to medium risk zone and enters into high risk zone.
Case 2: Vehicles travelling away from the PAS, means that the vehicle is moving from high risk zone to medium risk zone and enters into low risk zone.

The travel direction of the vehicle is computed by using the formula:

$$TD={Vpos}_{t}{-Vpos}_{t-1}$$ (1)

Where TD is the vehicle’s travel direction, VPos is the vehicle’s position in the highway and ‘t’ is time. If the TD value is negative then it is concluded that the vehicle is moving towards possible accident site(ie low risk zone to high risk zone)whereas if the TD value is positive then it is concluded that the vehicle is moving away from possible accident site(ie high risk zone to low risk zone).

#### Risk Factor Assignment

The Risk Factor (RF) assignment module assigns an RF value to the vehicles based on the identification of risk zone and travel direction. The RF value is assigned from0 to 3 for the vehicles in non, low, medium and high risk zones respectively. The value 3 is considered as the highest RF and 0 is considered as the lowest RF.

#### EWM Prioritization

This module assigns Reception Priority (RP) to the vehicles in the VBN structure. The vehicle which comes under *case1* (in travel direction (TD) identification section) should be given highest priority for EWM reception because the vehicle coming towards possible accident site may meet with an accident and alerting this vehicle holds the highest Reception Priority (RP) as 2. On the contrary in *case2* (in travel direction (TD) identification section), the vehicles which are traveling away from possible accident site are not going to meet with the accident but they can be used to alert the vehicles in medium and low risk zones. Hence these vehicles are assigned the reception priority as 1. The EWM should be instantly disseminated to the vehicles whose reception priority value is 2 and then to the vehicles whose reception priority value is 1.

### Performance Metrics

The performance of the RAP is decided based on the parameters such as S-D distance, EWM notification and end-to-end delay during EWM dissemination. Hence more care is taken in RAP scheme to (i) reduce the S-D distance (ii) increase the EWM notification and (iii) reduce the end-to-end delay.

**Fig 10:**
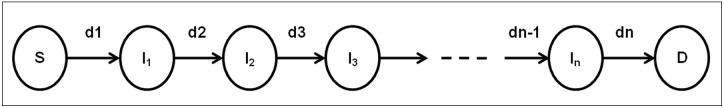
Specification of S-D distance.

The S-D distance in the VBN structure is the distance between the source RSU and the destination vehicles as shown in Fig 10.

The S-D distance can be computed by using the formula:

$${SD}_{distance}=\sum_{i=1}^{n}d_{i}$$ (2)

Where, S is the source RSU, D is the destination vehicle, n is the total number of intermediate nodes, I_i_ is the intermediate relay nodes and d_i_ is the distance between a pair of nodes in the S-D path. As explained in vehicular backbone network (VBN) formation phase section, the S-D distance is mostly reduced due to the utilization of VBN structure than the traditional approach.

The Notification is defined as the ratio between the number of vehicles notified with EWM and the total number of vehicles participating in the VBN structure.

The Notification Percentage (NP) is computed as:

$$NP=\frac{VN}{TV}x\;100$$ (3)

Where, VN is the number of EWM notified vehicles and TV is the total number of vehicles in the VBN structure.

The end-to-end delay in a network is defined as time taken for a message or a packet to reach the desired destination from the source. The end-to-end delay is calculated as,

$$D_{end-end}=Nx({d_{trans}+d}_{prop}{+d}_{proc})$$ (4)

Where, *D*_*end-end*_is the end-to-end delay, *N* is the total number of links in S-D path, *d*_*trans*_ is transmission delay, *d*_*prop*_ is propagation delay and *d*_*proc*_is called processing delay. The queuing delay is not considered in expression (4) due to critical nature of the EWM, it cannot be queued and kept in waiting. During simulation, the queuing delay is not noticed.

In addition, the metrics such as EWM notification time, reception rate, network processing overhead and notification distribution are also considered for simulation and are explained in section results and discussion.

### Simulation Setup

The Network Simulator-2 (version 2.34) is used for simulation to assess the performance of RAP schemes (RAP scheme1 and RAP scheme2). The simulation is done on the linear highway scenario in VANET. To accomplish this highway scenario, Freeway Mobility (FM) model is used. The Freeway Mobility model generates mobility of vehicular nodes in the freeway. A freeway is a highway without any obstruction such as traffic signals, intersections (or) crossings. Other roads and railways cross these freeways using either over bridge or under way [61].The main parameters used for simulation are listed in Table 7.

**Table 7:** Simulation Parameters.

PARAMETER	VALUE
Highway Segment Size	1000 meters
Number of Lanes	4 Lanes (2 in each direction
Mobility Model	Freeway Mobility Model
MAC Protocol	IEEE 802.11p (2Mbps)
Vehicle Speed Limits	60 km/h–90 km/h
Vehicle Density	20 to 160 in 500 meters
Number of Vehicles	200 in 1000 meters
Number of Sources	1 (RSU)
Coverage of Source RSU	500 meters
Coverage of the Vehicle	200 meters
Traffic Injection Rate	(1/75, 1/60, 1/45, 1/30, 1/15, 1/10) Vehicle/Second/Lane
EWM Notification Range	700 meters
Period of Message Exchange	100 milliseconds (ie 0.10 s)
Simulation Time	300 seconds

In simulation, two types of nodes are used. First, the RSUs (including Source) are set as fixed or stationary nodes. Second, traveling vehicles are set as mobile nodes. The simulation is performed involving all the four lanes (two lanes in each direction) of the highway road segments.

During simulation experiment, the vehicular nodes are injected into four lane highway road segment at different intervals to maintain heterogeneous traffic with different inter-distances among the vehicular nodes. For example, the traffic injection rate 1/10 means that a vehicular node is injected into a lane of the highway road segment once in every 10 seconds and this is called traffic injection rate. Similarly, various traffic injection rates are used in the simulation experiment such as 1/75, 1/60, 1/45, 1/30 and 1/15.

The behavior of the vehicles in the highway roads such as sudden stoppage, lane change, overtaking, acceleration and deceleration can be accomplished by applying the FM model. By varying the vehicle injection rate, the vehicle density can be varied from dense to sparse and vice-versa. For better simulation, the vehicles are moved at random locations by adjusting the vehicle injection rate and travel speed of the vehicles. The vehicular mobility patterns for RAP scheme1 (without VBN structure) and RAP scheme2 (with VBN structure) are generated by using the FM model. These mobility patterns are feed to NS-2 for simulation.

During simulation runs, abnormal values are set to certain vehicles to behave against smooth mobility in the highway road lanes. These vehicles are the root causes of road traffic accidents. These vehicles are set to perform abnormal behaviors such as sudden stoppage, abnormal lane change, abnormal acceleration and abnormal deceleration. The source node (RSU) is set to monitor this abnormality and react accordingly. The behavior and working of the source, moving vehicles and relay vehicles are clearly specified in simulation settings. Generation of traffic mobility patterns, creation of abnormality and EWM generation are done separately for RAP scheme1 and RAP scheme2. The coverage of source (RSU) is fixed as 500 meters and attempt is made to extend this to 700 meters by using the VBN structure.

## Results and Discussion

The simulation results show that the RAP scheme performs well in prevention of highway road traffic accidents. Two types of RAP schemes are simulated such as, (i) RAP scheme1 (without VBN structure) and (ii) RAP scheme2 (with VBN structure). The results demonstrate that RAP scheme2 outperforms RAP scheme1 and improves the performance of EWM dissemination using the VBN structure in VANET.

To assess the performance of RAP schemes, the following metrics are used:

Notification: Number of vehicles successfully notified with EWMs by the source (RSU) against the total number of vehicles in the VBN structure in percentage.

End-to-end delay: The time taken for EWMs to reach the desired destinations from the source (RSU). The end-to-end delay includes transmission, propagation and processing delays for all individual links between pair of nodes in the transmission path. Queuing delay is not considered because the EWMs need to be instantly disseminated.

Reception rate: It is the percentage of vehicles that intend to receive the EWM successfully.

Network processing overhead: It is the number of messages used by the network for the processes such as RZ identification, TD identification, RF assignment and EWM prioritization.

Notification Distribution: It is the number of EWM notifications across HRZ, MRZ and LRZ zones.

### Effects of change in Vehicle Density (VD)

The simulation is done to assess the performance of RAP scheme with respect to change in Vehicle Density (VD). Separate simulation runs have been done to assess all the performance metric and the results are recorded and analyzed. Both RAP scheme1 and RAP scheme2 are simulated for various vehicle densities to analyze the results. The observations made during simulation are discussed below.

#### Notification

Initially, 20 vehicles are made to move in the network (highway roads) with different injection rates. Based upon abnormal prediction report, the source (RSU) node generates EWM and disseminates it to these vehicles and the response is noticed. During the stipulated period (100ms), the RAP Schemes1 made only 17 successful notifications whereas the RAP Scheme 2 came out with 18 successful notifications. In this way, the vehicle density has been gradually increased to 160 vehicles and the notification has been recorded for both RAP schemes in different simulation runs.

**Fig 11:**
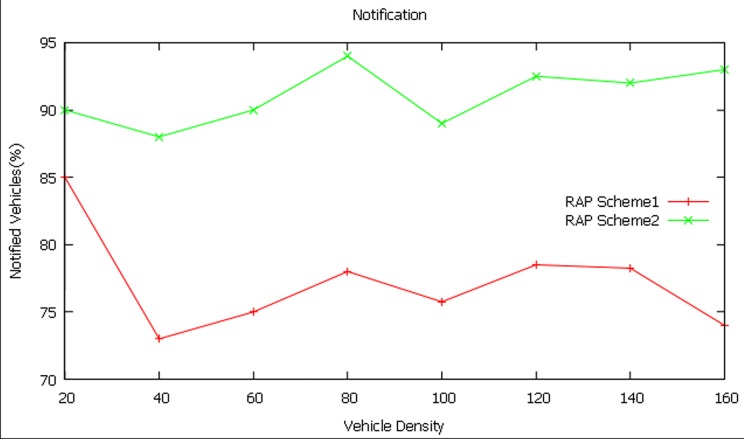
Notification at different vehicle densities.

The notification for a vehicle density of 160 vehicles is found to be 74 percent and 93 percent for RAP scheme1 and RAP scheme2 respectively as shown in Fig 11. It is observed from the simulation runs that the RAP scheme2 performs better than RAP scheme1 by 19percent for the vehicle density of 160 vehicles. From simulation, it is found that the average notification of RAP scheme2 is 14 percent more than that of the RAP scheme1.

#### End-to-End Delay

The simulation is done in the same way to identify end-to-end delay in the network with varied vehicle densities (20 to 160).

**Fig 12:**
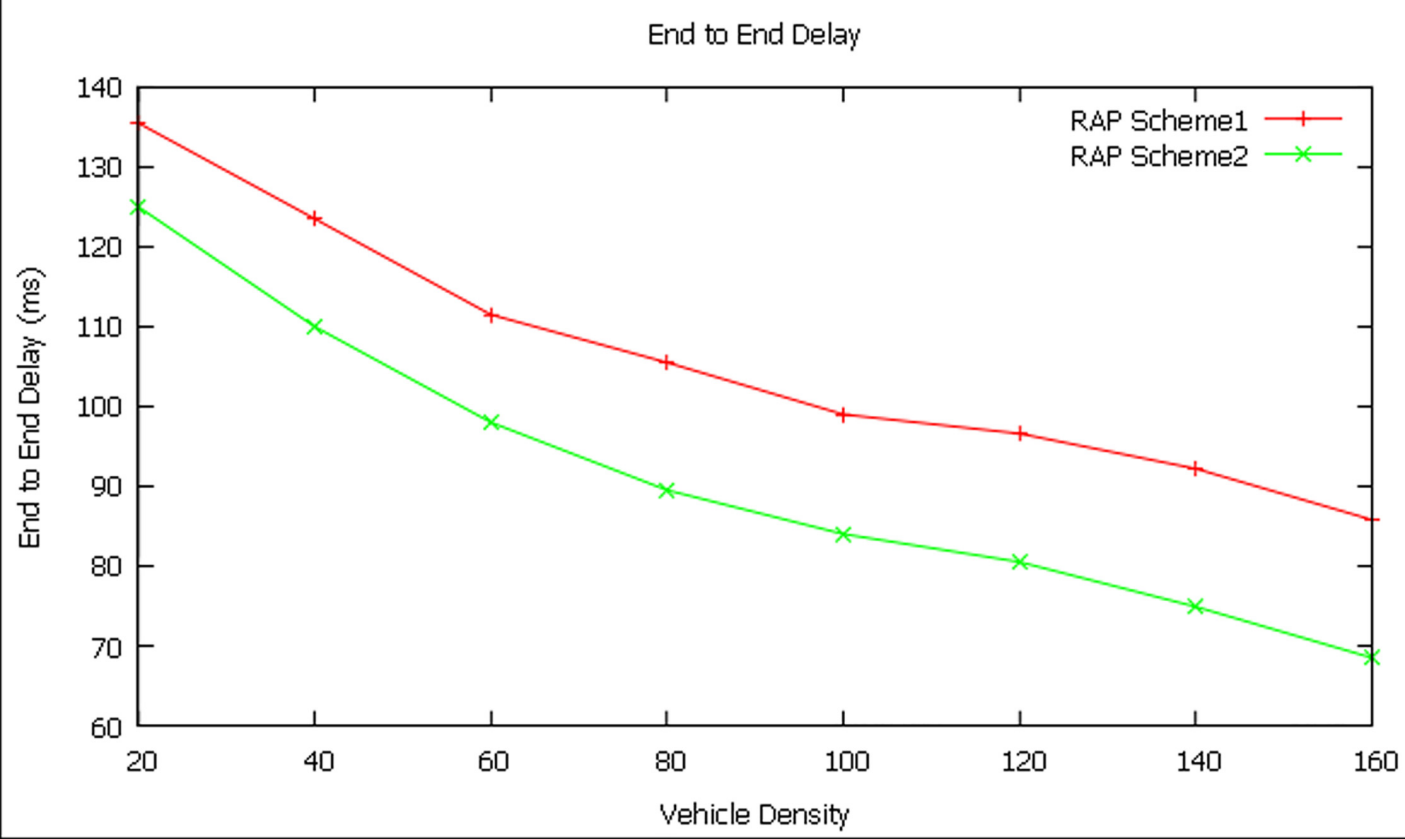
End-to-end delay at different vehicle densities.

Fig 12 shows that for 20 vehicles, the end-to-end delay is found to be 138 ms and 130 ms for RAP scheme1 and RAP scheme2 respectively. On the contrary, the end-to-end delay is found to be 78 ms for RAP scheme1 and 55 ms for RAP scheme 2 for the vehicle density of 160 vehicles.

In case of end-to-end delay, it is noticed that the delay is more if the VD is lesser and the delay is less if the vehicle density is larger. This shows that if the vehicles in the VBN structure are sparse then the delay is higher because the S-D distance is more. On the other side, if the vehicles in the VBN structure are denser then the delay is lower due to lesser S-D distance.

The RAP scheme2 provides better performance than RAP scheme1 in terms of end-to-end delay by 14.38 percent for vehicle density of 160 vehicles.

Initially, for a vehicle density of 20, the end-to-end delay is recorded as 125 ms and this gradually decreases across different vehicle densities and is found as 68.5 milliseconds for vehicle density of 160. It is proved from the simulation experiment that the end-to-end delay decreases with respect to increase in vehicle density. The simulation experiment has been done for a vehicle density up to 160. Further, based on the result, it can be concluded that if the vehicle density is increased beyond 160 then end-to-end delay will certainly decrease. In a dense VBN structure, due to the lesser source-destination distance, the end-to-end delay is found to be minimum than in sparse VBN structure.

#### Reception rate

**Fig 13:**
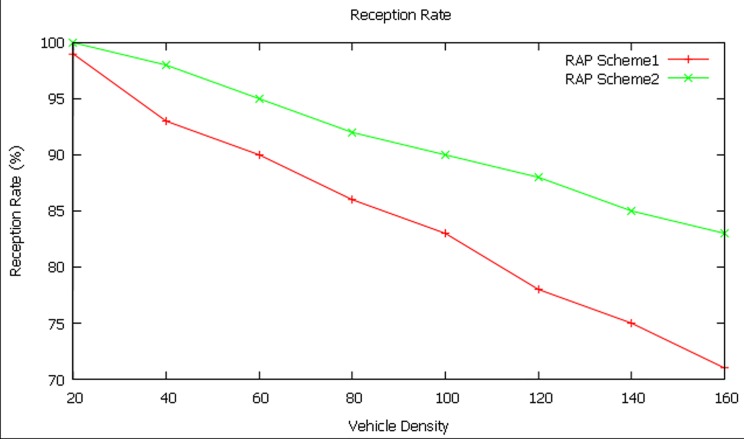
Reception rate at different vehicle densities.

In this case, the increase in vehicle density makes reception rate to decrease as shown in Fig 13. This may be because of problems such as increase in interference, collision due to hidden nodes, etc.

The RAP scheme 2 achieves an average reception rate of 91.37 percent which is better than the average reception rate of 84.37 percent of RAP scheme 1. This shows that the performance of RAP scheme 2 is more by 7 percent when compared to RAP scheme 1.

#### Network processing overhead

**Fig 14:**
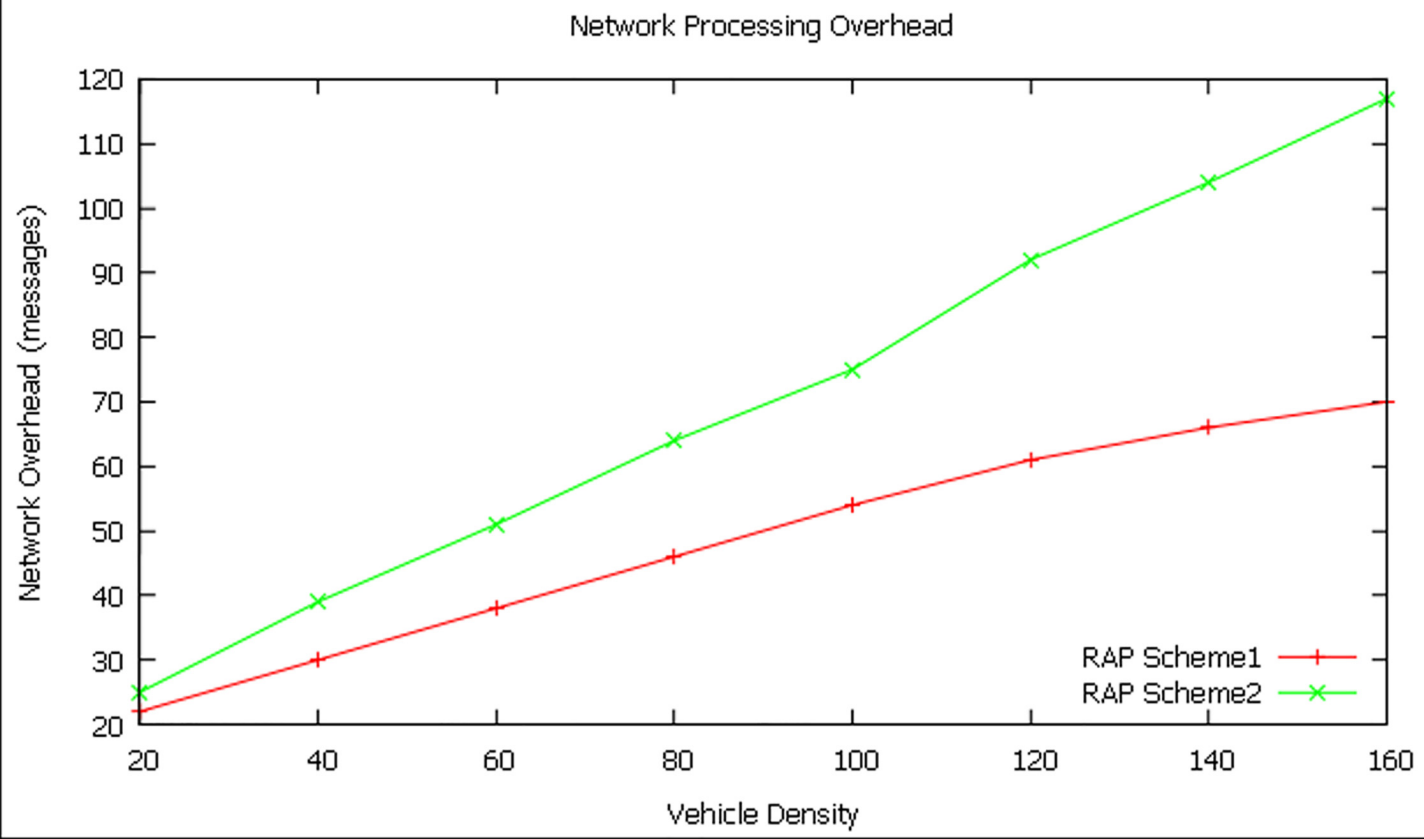
Network processing overhead at different vehicle densities.

Fig 14 shows that the network processing overhead is higher in RAP scheme2 when compared to RAP scheme1. This is because of the additional messages transmitted (excess time taken) by the RAP scheme2 for processes such as RZ identification, TD identification, RF assignment and EWM prioritization processes.

The RAP scheme2 lacks in Network Processing Overhead by27.68 percent when compared to the RAP scheme1.

#### Notification Distribution

**Fig 15:**
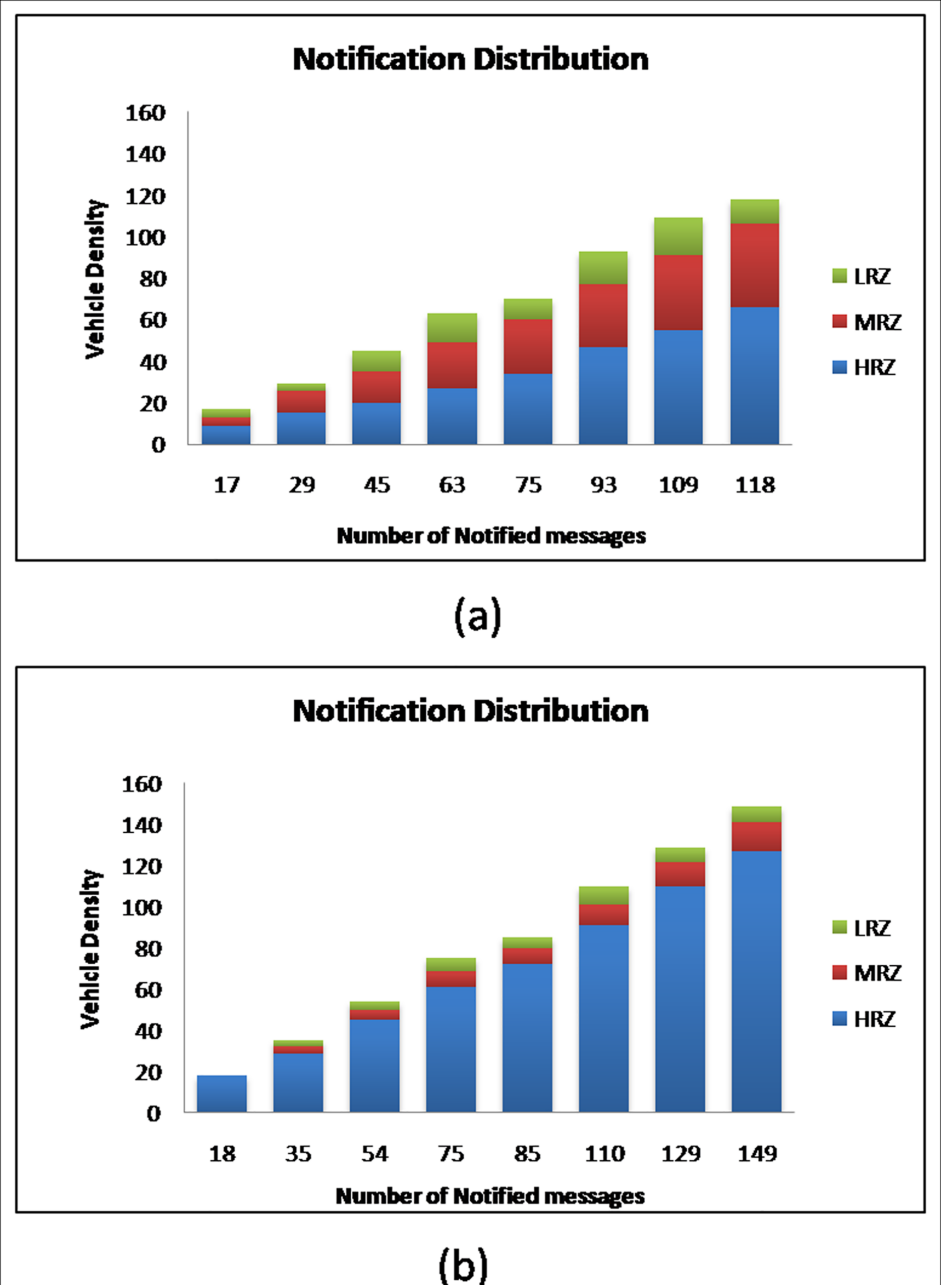
Notification Distribution for (a) RAP Scheme 1 (b) RAP Scheme 2.

It is observed from simulation that the EWMs are successfully notified / disseminated to most of the vehicles in HRZ and in the area of high risk factor by RAP scheme2 than RAP scheme1. The notification distribution for RAP scheme1 and RAP scheme2 against various vehicle densities is shown in Fig 15A and 15B respectively.

It is clear from the above observation that the RAP scheme2 outperforms RAP scheme1 by performing EWM dissemination to most of the vehicles in HRZ than to the vehicles in MRZ and LRZ. The RAP scheme2 disseminates 85.70 percent of EWMs to the vehicles in HRZ and the remaining EWMs to MRZ and HRZ zones. On the contrary, the RAP scheme1 disseminates only 49.27 percent of EWMs to the vehicles in HRZ.

Further, it is observed from the simulation that the performance of RAP scheme2 is low if the vehicle density is lesser than 40 and the performance is promising for vehicle density greater than 40. This means that the RAP scheme2 performs well in a dense VBN structure than a sparse VBN structure.

### Effects of change in Highway Road Lanes

In addition to the change in vehicle density, the performance of RAP scheme 2 (with VBN structure) is also analyzed for different highway roads with 2-lane, 4-lane and 6-lane. Throughout the simulation experiment, the vehicle density is initialized to 20 and it is gradually increased through 160. The simulation experiments are carried out separately to assess all the performance metrics and the results are analyzed. The observations made during simulation experiments are discussed below.

#### Notification

**Fig 16:**
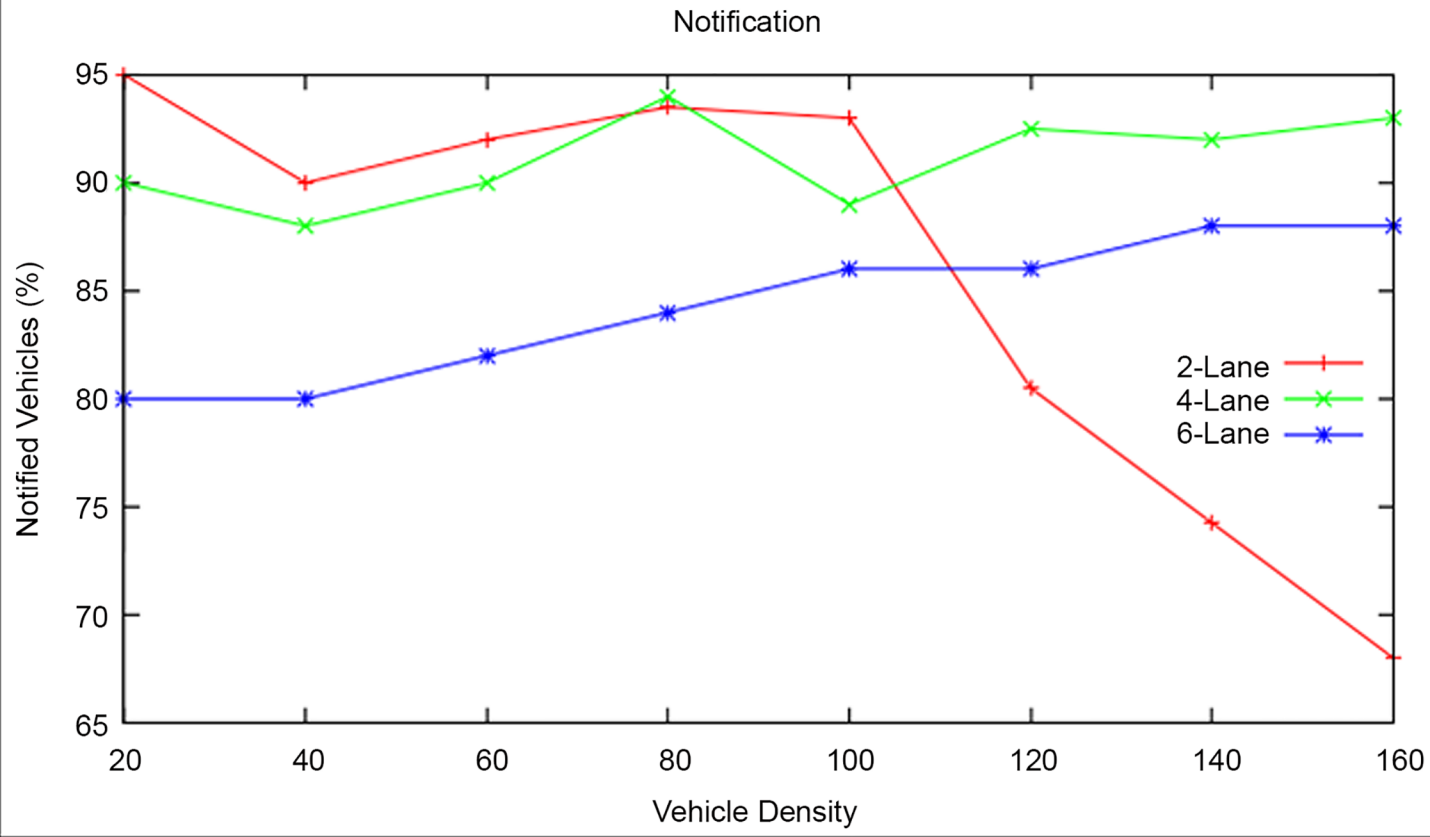
Notification at different Vehicle Densities and Highway Road Lanes.

During the specified time period, for a vehicle density of 20, the RAP scheme came out with 19, 18, 16 notifications in 2-lane, 4-lane, 6-lane highway roads respectively. For a vehicle density of 160, the RAP scheme came out with 110, 149, 144 notifications in 2-lane, 4-lane, 6-lane highway roads as shown in Fig 16.

The average notification of RAP scheme for various vehicle densities is found to be 86.32 percent, 91 percent, 84.25 percent in 2-lane, 4-lane, 6-lane highway roads respectively. It can be clearly observed that the notification performance of the RAP scheme is 4.68 percent and 6.75 percent high in 4-lane highway road than in 2-lane and 6-lane highway roads. It is noticed that the notification degrades beyond the vehicle density of 100 in 2-lane highway road. It is also noticed that the notification is promising in 4 lane highway roads than others. It is further noticed that the notification in 6-lane highway road gets increased across various vehicle densities.

#### End to End Delay

**Fig 17:**
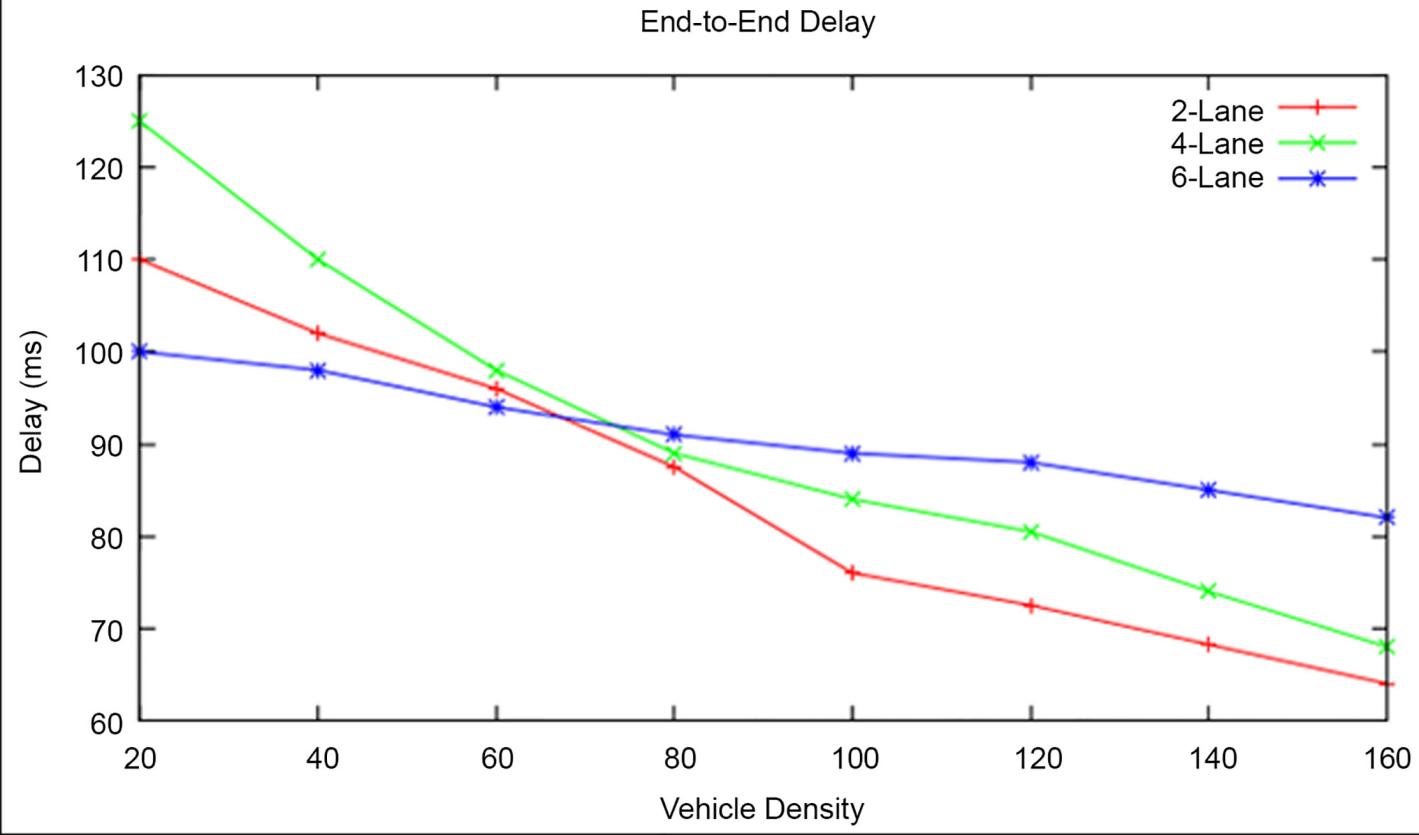
End-to-End Delay at different Vehicle Densities and Highway Road Lanes.

The end-to-end delay for a vehicle density of 20 is found to be 110ms, 125ms, and 145ms in 2-lane, 4-lane and 6-lane highway roads respectively and the same for a vehicle density of 160 is found to be 64ms, 68ms, 125 ms in 2-lane, 4-lane and 6-lane highway roads respectively as shown in Fig 17.

The simulation experiment shows that the end-to-end delay gradually decreases with respect to increase in vehicle density in 2-lane and 4-lane highway roads. On the contrary, the end-to-end delay slightly decreases in the 6-lane highway road with respect to increase in vehicle density. The end-to-end delay is very high in 6-lane highway road than the other two types. The results clearly prove that the end-to-end delay is lesser in a dense highway road than in a sparse highway road. If the vehicle density is further increased beyond 160 then the end-to-end delay in the 6-lane highway road will get decreased.

#### Reception Rate

**Fig 18:**
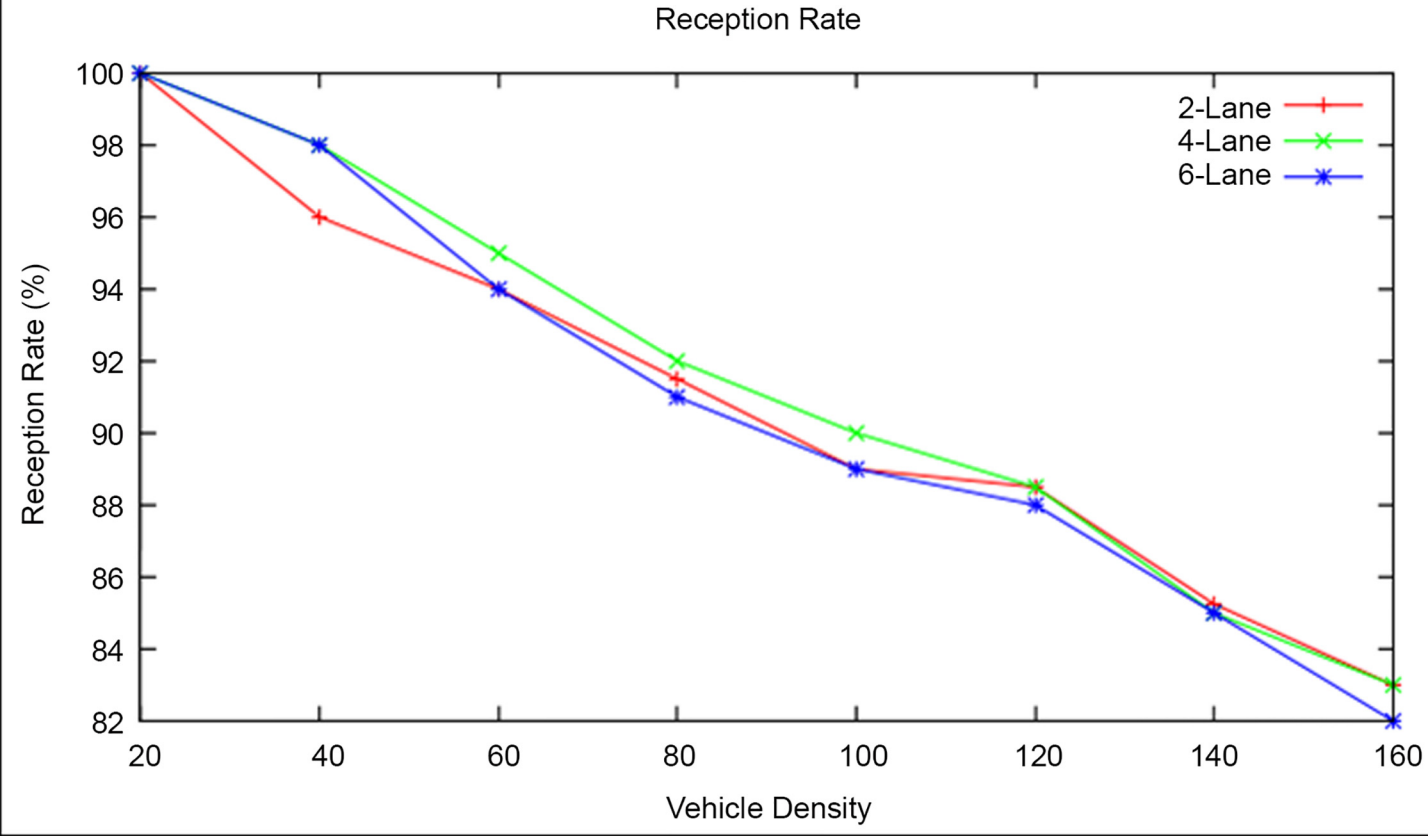
Reception Rate at different Vehicle Densities and Highway Road Lanes.

Initially for a vehicle density of 20, the reception rate of the vehicles is found to be 100 percent. But the reception rate decreases with respect to an increase in the vehicle density for all types of highway road lanes. As mentioned in section effects of change in vehicle density, the problems such as interference or collision due to hidden nodes may be the root cause of decrease in reception rate. The average reception rate of RAP scheme is found to be 90.7 percent, 91.37 percent and 91 percent for the 2-lane, 4-lane and 6-lane highway roads respectively as shown in Fig 18. This result shows that the reception rate in the 4-lane highway road is slightly better than the other two.

#### Network Processing Overhead

**Fig 19:**
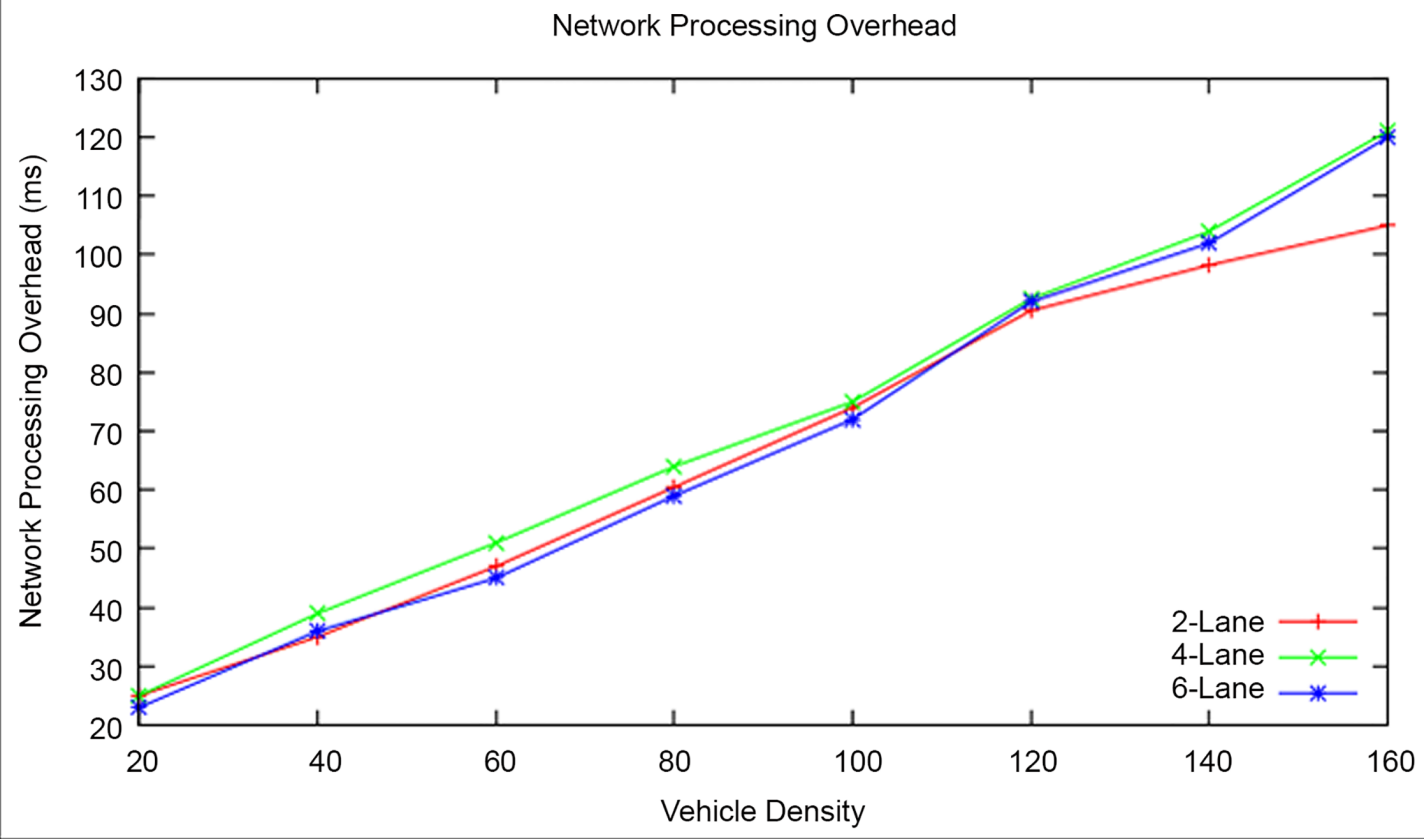
Network Processing Overhead at different Vehicle Densities and Highway Road Lanes.

The network processing overhead increases proportionally with an increase in vehicle density for all types of highway road lanes as shown in Fig 19. The network processing overhead in the RAP scheme is higher because of the additional messages incurred for the tasks such as risk zone identification, travel direction identification, risk factor assignment and EWM prioritization. The network processing overhead is less for 2-lane highway road when compared to other types of the highway roads.

#### Notification Distribution

**Fig 20:**
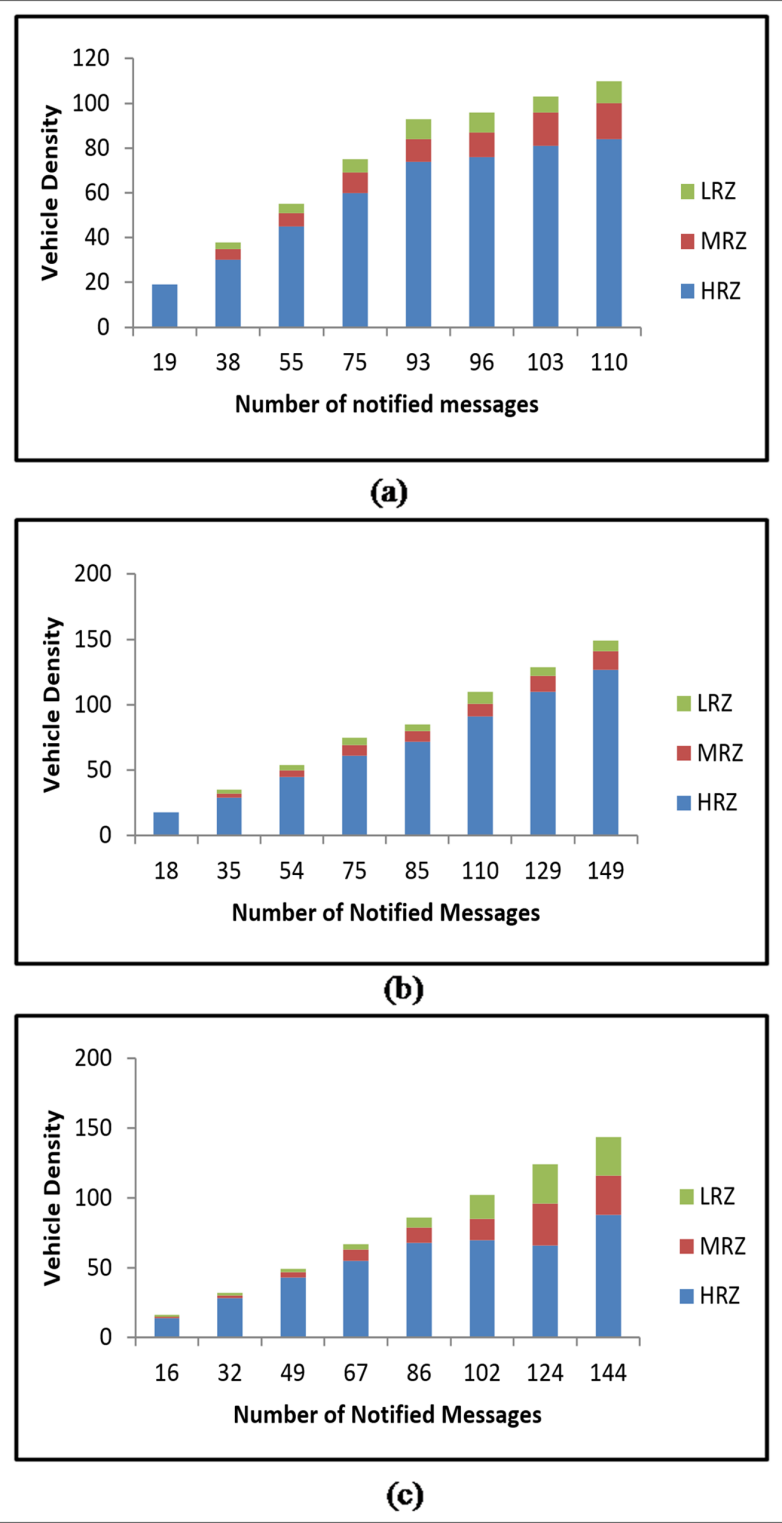
Notification Distribution at different Vehicle Densities in (a) 2-Lane (b) 4-Lane (c) 6-Lane Highway Roads.

The prime objective of the RAP scheme is to deliver EWM to the vehicles that have a high risk factor and travel in high risk zone. The results of the simulation experiment show that the EWMs are mostly distributed to the vehicles that travel in high risk zone in all three types of highway road lanes. But the notification distribution in 4-lane highway road is found better than that of the other two. This can be noticed from the results plotted in Fig 20. In 4-lane highway road, 149 EWMs are notified to the vehicles. Out of them, 127 EWMs are notified to the vehicles in high risk zone. This is far better against other two types of highway road lanes.

During simulation, the following observations are also made:

1. The simulation results make it clear that if the highway road segment is sparser then the end-to-end delay is higher. In case if the highway road segment is denser then the end-to-end delay is lower. This is clearly reflected in the notification of the RAP schemes.
2. It is noticed that upon detection of abnormal prediction report, the RAP scheme2 responds for EWM notification to the vehicles at an average of 93ms for vehicle densities 20 through 160.
3. The performance of RAP scheme2 is promising for a single abnormal event (Road Traffic Accident in PAS) whereas the performance degrades for two abnormal events and so on. This means that if more than one accident takes place within the coverage area of the RSU then the performance of RAP scheme2 is degrading. It is because the RSU has to undergo the steps involved in RAP scheme2 within the stipulated time period. Hence, if more number of accidents take place the performance is slightly degraded.
4. The DISMN timer is reduced from 100ms to 75ms and the reactions are noticed during simulation. The performance of RAP scheme2 degrades by delivering only lesser number of EWMs.
5. During a simulation run, the system fails to deliver EWM to all the vehicles in the HRZ and in the area of high RF. This is because of the failure of relay node in the VBN structure.
6. From simulation, it is noticed that the overall performance of RAP scheme2 is 95 percent in terms of success. This is recorded based on the successful EWM disseminations of RAP scheme2 during 20 simulation runs.
7. The RAP scheme with VBN structure shows better performance in 4-lane highway road than 2-lane and 6-lane highway roads.
8. The network processing overhead in 2-lane highway road is found to be better than 4-lane and 6-lane highway roads.
9. The End-to-end delay is much higher in 6-lane highway road than 2-lane and 4-lane highway roads.
10. The EWM notification distribution in 2-lane highway road is much promising than 4-lane and 6-lane highway roads.

The overall performance of RAP scheme with VBN structure is better than RAP scheme without VBN structure in terms of notification, end-to-end delay, reception rate and notification distribution. However, the network processing overhead is higher in RAP scheme with VBN structure when compared to RAP scheme with VBN structure. This is because of the additional number of steps involved in VBN formation and EWM dissemination processes. In addition, it is proved from the simulation experiment that the RAP scheme with VBN structure performs well in 4-lane highway roads than 2-lane and 6-lane highway roads.

## Conclusion and Future Work

In this paper, a Road Accident Prevention (RAP) scheme for instant EWM dissemination to the vehicles is proposed in order to prevent them from highway road traffic accidents. Thereby the death and injury rates can be reduced in Indian four lane highways. In RAP scheme, once the RSU predicts the possibility of occurrence of an accident or emergency situation, instantly it generates EWM, forms a VBN structure and disseminates EWM to the vehicles which have high reception priority. The performance evaluation of RAP schemes is done by using NS-2 simulator. From the simulation results, it is noticed that RAP scheme with VBN structure outperforms RAP scheme without VBN structure by providing better EWM dissemination performance in terms of (i) reducing the S-D distance, (ii) improving notification by 19 percent and (iii) reducing end-to-end delay by 14.38 percent. Further, the number of RSUs required is reduced due to the usage of VBN structure in VANET. But, the network processing overhead of RAP scheme with VBN structure is found to be higher. In future attempts can be made to reduce this overhead. It is proved from the simulation experiment that the overall performance of the RAP scheme is promising in four lane highway road than its counter parts such as two lane and six lane highway roads. The impacts of the RAP scheme will be analyzed based on fuel consumption and emissions of the traffic flow in future. The RAP scheme is designed only for linear four lane express highways. In future, the RAP scheme can be enhanced to work with real two dimensional highways.
